# Viral metagenomics reveals diverse virus-host interactions throughout the soil depth profile

**DOI:** 10.1128/mbio.02246-23

**Published:** 2023-11-30

**Authors:** George Muscatt, Ryan Cook, Andrew Millard, Gary D. Bending, Eleanor Jameson

**Affiliations:** 1 School of Life Sciences, University of Warwick, Coventry, United Kingdom; 2 School of Veterinary Medicine and Science, University of Nottingham, Loughborough, United Kingdom; 3 Department of Genetics and Genome Biology, Leicester Centre for Phage Research, University of Leicester, Leicester, United Kingdom; 4 School of Natural Sciences, Bangor University, Bangor, Gwynedd, United Kingdom; University of California, Irvine, California, USA

**Keywords:** antagonistic co-evolution, bacteriophages, lysogeny, macrodiversity, microdiversity, positive selection, soil depth, virus-host interactions

## Abstract

Soil microbes play pivotal roles in global carbon cycling; however, the fundamental interactions between microbes and their infecting viruses remain unclear. This is exacerbated with soil depth, where the patterns of viral dispersal, ecology, and evolution are markedly underexplored. To investigate viral communities throughout the soil depth profile, we leveraged a publicly available metagenomic data set sampled from grassland soil in Northern California. In total, 10,196 non-redundant viral operational taxonomic units were recovered from soil between 20 cm and 115 cm below the surface. Viral prevalence was high throughout the soil depth profile, with viruses infecting dominant soil hosts, including *Actinomycetia*. Contrary to leading hypotheses, lysogeny did not dominate in the soil viral communities. Viral diversity was assessed at both the population level (i.e., macrodiversity) and strain level (i.e., microdiversity) to reveal diverse ecological and evolutionary patterns of virus-host interactions in surface and subsurface soils. Investigating viral microdiversity uncovered potential patterns of antagonistic co-evolution across both surface and subsurface soils. Furthermore, we have provided evidence for the potential of soil viruses to augment the remineralization of soil carbon. While we continue to yield a more comprehensive understanding of soil viral ecology, our work appeals to future researchers to further investigate subsurface viral communities.

**IMPORTANCE:**

Soil viruses can moderate the roles that their host microbes play in global carbon cycling. However, given that most studies investigate the surface layer (i.e., top 20 cm) of soil, the extent to which this occurs in subsurface soil (i.e., below 20 cm) is unknown. Here, we leveraged public sequencing data to investigate the interactions between viruses and their hosts at soil depth intervals, down to 115 cm. While most viruses were detected throughout the soil depth profile, their adaptation to host microbes varied. Nonetheless, we uncovered evidence for the potential of soil viruses to encourage their hosts to recycle plant-derived carbon in both surface and subsurface soils. This work reasons that our understanding of soil viral functions requires us to continue to dig deeper and compare viruses existing throughout soil ecosystems.

## INTRODUCTION

Soil microbes are integral members of terrestrial ecosystems, with microbial metabolism contributing to global carbon cycling (1). As obligate parasites of microbes, viruses can control their hosts’ population size through lytic infections and influence their hosts’ metabolic potential through the expression of auxiliary metabolic genes (AMGs) (2–6). In the oceans, where virus-host interactions have been more thoroughly studied, viral lysis is estimated to turnover ~20% of microbial biomass each day (7). The subsequent liberation of dissolved carbon and nutrients increases microbial respiration and limits trophic transfer up the food web (8, 9). Despite an appreciation for the ecological roles of viruses in marine ecosystems, the relevant functions of viruses in terrestrial ecosystems have received less attention. To resolve this, recent methodological developments have provided the means to investigate soil viral ecology through metagenomics (10–12), and we are beginning to uncover the ecosystem-level impacts of soil viruses (13).

Integral to understanding soil viral ecology are the fundamentals of viral dispersal, prevalence, and persistence. The consequence of these factors is demonstrated by the structuring of viral communities across gradients of space (14–18), time (13, 16, 17), and root/soil compartment (13, 19). However, most ecological studies have focused on surface soils, rendering subsurface viral communities markedly underexplored. This is particularly alarming given the disparity in soil biogeochemistry between surface and subsurface niches. For example, more than half of terrestrial carbon stocks are sequestered in subsurface soils (20), with microbial respiration and biomass turnover dictating long-term carbon storage (21, 22). Additionally, subsurface microbial communities are key drivers of pollutant biodegradation, thus controlling their fate and dispersal to groundwater resources (23). Given the pressures of viral infection on the mortality and metabolism of host populations, investigations into subsurface soil viral ecology could inform global actions for mitigating climate change and promoting bioremediation.

Numerous physicochemical properties of soil vary throughout its vertical profile (24, 25). These factors shape the distribution of microbial populations such that community variation with depth is comparable to the variation observed between surface soils from different biomes (26). Thus, the structuring of microbial communities may reflect variation in microbial responses to nutrient availability between ecological niches. Given the requirement of host cellular machinery for replication and the specificity of host infection, the structuring of viral communities is likely to be highly dependent on that of their host community. Subsequently, there is great importance in characterizing the repertoire of fundamental virus-host interactions.

Amid exponentially decreasing host biomass, activity, and diversity in subsurface soil (26, 27), virus-host interactions are likely to vary considerably with depth. For example, microscopic investigations have found that the virus-to-bacteria ratio decreases with soil depth (28). Lower virus-to-bacteria ratios have been associated with an increased prevalence of lysogeny (28, 29), a latent infection strategy where the viral genome replicates passively within the host’s chromosome until being induced. Lysogenic infections can have significant impacts on the ecology and evolution of their host communities (hereafter referred to as “eco-evolutionary interactions”) (30). While temperate viruses, capable of lysogeny, have been predicted to dominate in soils (31–33), relevant metagenomic studies have failed to corroborate this (12, 13, 34). The argument for such increased lysogeny, namely, the reduced access to viable hosts (28, 29, 35), is even more profound in subsurface soil. Therefore, further studies investigating subsurface viruses are required to understand infection strategy preferences in soils.

The co-evolution of viruses and their hosts contributes to the emergence and maintenance of phenotypic diversity in both partners (36–38). This relationship is inherently antagonistic since the adaptation of one partner puts the survival of the other at a disadvantage. However, we understand very little about *in situ* antagonistic co-evolution and even less across environmental gradients such as soil depth. Given the stark differences in nutrient availability over short vertical distances (24, 25), which have been evidenced to impact co-evolution dynamics (39), we hypothesize that the eco-evolutionary interactions between viruses and their hosts vary throughout the soil depth profile. This is likely to implicate soil viruses in the soil major biogeochemical processes, as has been demonstrated for marine ecosystems (8, 40).

In this study, we leveraged a publicly available metagenomic data set assembled from Californian grassland soil (41) to investigate viral communities between 20 cm and 115 cm below the soil surface. Grasslands cover ~40% of non-glacial land area (42), store a third of global terrestrial carbon (43), and provide numerous ecosystem services from food production to erosion regulation (44). Moreover, a quantitative stable-isotope probing approach was recently used to associate the activities of viruses and their hosts in grassland soil (45). Therefore, grassland ecosystems are an ideal model system for investigating the eco-evolutionary interactions between soil viruses and their microbial hosts. Two soil depth profiles were sampled, representing contrasting aboveground vegetation: under a Garry oak tree (“Garry Oak” samples) versus neighboring grassland (“Hilly grassland” samples). To uncover patterns of viral dispersal, ecology, and evolution throughout soil depth, we assessed viral diversity at both the population level (i.e., macrodiversity) and strain level (i.e., microdiversity). This study aimed to answer the following questions: (i) To what extent does soil depth shape the assembly of viral communities, and is this effect consistent between sites? (ii) Does lysogeny vary throughout the soil depth profile, such that temperate viruses dominate in subsurface soil? (iii) How do the eco-evolutionary interactions between viruses and their hosts vary throughout the soil depth profile?

## MATERIALS AND METHODS

### Field site

Soil was sampled previously (41) at the Sagehorn study site within the Eel River Critical Zone Observatory in Northern California. The site is underlain by the Central Belt of the Franciscan Formation, a mélange of sheared argillaceous matrix containing blocks of sandstone and other lithologies (46). The soil profile comprises a surface organic-rich horizon (~30 cm) underlain by a clay-rich horizon (10 cm–20 cm), directly above saprolite (47). As a result of the low-porosity bedrock, the critical zone layers become entirely saturated during the winter wet season (47). Sagehorn is primarily a grassland ecosystem, with scattered Garry oak (*Quercus garryana*) trees. The region has a Mediterranean climate, described by hot, dry summers (from May to September) and cool, wet winters. The average rainfall for the region is ~1,800 mm, with 1,976 mm of precipitation recorded during the year that soil samples were taken (47).

### Sample collection

The collection of soil samples was previously performed at the Sagehorn study site in Northern California in June 2016, by Sharrar et al. (41). The vertical soil depth profile was sampled in duplicate at 20 cm, 40 cm, 60 cm, 80 cm, 100 cm, and 115 cm. Soil pits were dug using a jackhammer, and the walls of the pits were sampled on both sides with a sterile scoop, resulting in two samples per soil depth collected approximately 10 cm apart laterally. Soil profiles was sampled at two sites: under a Garry oak tree (“Garry oak” samples) and from the grassland approximately 10 m away (“Hilly grassland” samples), for a total of 24 samples.

### Metagenomic data set access

The metagenomes assembled from each soil sample described above were accessed from NCBI under project accession PRJNA577476 (sample accessions SAMN13153360-SAMN13153383).

### Recovery of viral populations

Viral contigs were predicted from the pooled assembled metagenomes (PRJNA577476). Double-stranded DNA (dsDNA) and single-stranded DNA (ssDNA) viral contigs ≥ 5 kilobase pairs (kb) were predicted with DeepVirFinder v1.0 (48), VIBRANT v1.2.1 (49), and VirSorter v2.2.3 (50), using permissive viral score thresholds where relevant (≥ 0.8 for DeepVirFinder and ≥ 0.5 for VirSorter). The quality of the viral contigs predicted by any one of the three tools was assessed with CheckV v0.8.1 (51), and resulting trimmed viral sequences were annotated with DRAM v1.3 (52). Annotated viral sequences were then manually curated following the selection criteria outlined by Guo et al. (53). Briefly, sequences were confirmed to be of viral origin based on the presence of confident viral hallmark gene annotations, while sequences were deemed non-viral when containing cellular (e.g., plasmid-associated) annotations. In addition to these curated sequences, viral sequences with the most confident prediction scores from DeepVirFinder (with corresponding viral scores ≥ 0.95, *P ≤* 0.05, and length ≥ 10 kb) and from VIBRANT (with corresponding quality scores of “high quality draft” or “complete circular”, and length ≥ 10 kb) were also retained. All viral sequences were then clustered into viral operational taxonomic units (vOTUs) at 95% average nucleotide identity across 85% of the alignment fraction relative to the shorter sequence (54) using anicalc.py and aniclust.py scripts (51). This resulted in 10,196 vOTUs ≥ 5 kb, representing approximately species-level viral populations. Additional functional gene annotations were provided with Prokka v1.14.6 (55) using the Prokaryotic Virus Remote Homologous Groups (PHROGs) database (56).

To determine whether any recovered vOTUs represented previously isolated bacteriophage (phage) species, we clustered our vOTUs with the INfrastructure for a PHAge REference Database (INPHARED) of phage genomes (accessed February 2022) (57) using anicalc.py and aniclust.py scripts (51). Viral sequences were considered to represent the same species when they shared 95% nucleotide identity across 85% of the alignment fraction relative to the shorter sequence (54).

### Taxonomy of viral populations

Taxonomic assessment of vOTUs was achieved through shared protein clustering using vConTACT2 v0.9.22 (58) with the INPHARED phage genome database (accessed February 2022) (57) and otherwise default settings. The resultant genome network was visualized in R v4.0.5 (59) using ggnet2 from GGally v2.1.2 (60) and the Fruchterman-Reingold force-directed algorithm. Nodes (representing viral genomes) were connected by edges (representing shared protein homology), with significant connections forming viral clusters (VCs) representing roughly genus-level groups. Viral genomes sharing overlap with genomes from multiple VCs were considered as singletons. To further interrogate the similarity of recovered vOTUs to a database of > 600,000 environmental phage sequences, we used the web-based PhageClouds tool (61). By inputting the nucleotide sequences of our vOTUs, related environmental phage sequences from the database were identified using an intergenomic distance threshold of 0.21.

The phylogeny of jumbo phage vOTU and “jumbo-related” vOTU genomes was investigated using the DNA polymerase gene. The translated DNA polymerase gene sequences were queried against the INPHARED phage genomes database (accessed June 2022) (57) to identify closely related phage genomes using the ublast command from USEARCH v10.0.240 (62) and a similarity *E*-value threshold < 0.001. For downstream visualization, an outgroup of human alphaherpesvirus 1 was included in the analysis. The translated sequences of the DNA polymerase gene from the vOTUs and reference genomes were then aligned using MAFFT v7.271 (63, 64), with automated settings. Phylogenetic trees were constructed using IQ-TREE v1.6.3 (65, 66), the Whelan and Goldman protein substitution model, and 1,000 ultrafast bootstrap replicates (67). Trees were subsequently visualized in R using ggtree v2.5.3 (68–70).

### Characterization of viral populations

vOTUs were classified as temperate when they were identified by any of the three following methods. First, if the viral contig was excised from a flanking host scaffold by CheckV. Second, vOTUs carrying at least one gene associated with lysogeny (i.e., transposase, integrase, excisionase, resolvase, and recombinase) were considered temperate. Lysogeny-associated genes were identified using the Pfam domains: PF07508, PF00589, PF01609, PF03184, PF02914, PF01797, PF04986, PF00665, PF07825, PF00239, PF13009, PF16795, PF01526, PF03400, PF01610, PF03050, PF04693, PF07592, PF12762, PF13359, PF13586, PF13610, PF13612, PF13701, PF13737, PF13751, PF13808, PF13843, and PF13358, as previously described (71, 72). Third, vOTUs which formed a VC with at least one known temperate phage were also considered temperate.

Host assignment was achieved using a combination of methods. First, hosts were inferred using the microbial taxonomy assigned to the scaffold from which proviral sequences were excised from. Second, CRISPR spacers identified from assembled scaffolds using PILER-CR v1.06 (73) were used to identify complementary protospacers among vOTU genomes using BLASTn, with default settings and allowing for ≤ 2 mismatches. Additionally, CrisprOpenDB (74) was used with default settings. Lastly, host genera were predicted *de novo* using WIsH v1.0 (75) and a null model trained against 9,620 bacterial genomes, as previously described (71). Given that some vOTUs had conflicting host predictions between methods and that only a single host was considered per vOTU in our analyses, preferential assignment of hosts was ordered: provirus hosts > CRISPR spacer linkage to metagenome-assembled genome (MAG) > CRISPR spacer linkage to database genome > WIsH *de novo* prediction.

Putative viral-encoded AMGs were identified using DRAM-v (52). Due to the expected increased false positive signal arising from the high non-viral sequence space in the soil metagenomes, strict curation of candidate AMGs was performed, as suggested (76). Briefly, this included genes on viral contigs ≥ 10 kb or complete genomes, with an auxiliary score of 1–3 and with both the “M” flag (corresponding to metabolic function) and the “F” flag (corresponding to genes within 5,000 bases of the end of the viral contig).

AMGs encoding carbohydrate-active enzymes (CAZymes) were further interrogated for the detection of conserved functional domains using the Conserved Domain Search (CD-Search) service (77, 78). No CAZymes had the “A” flag from DRAM-v, which indicates tail association, implicating putative CAZymes as being involved in host metabolism instead of viral attachment.

### Abundance of viral populations

vOTU abundance was estimated by mapping raw metagenome reads against vOTU genomes using BBMap (79) with a minimum alignment identity of 90%. vOTUs were only considered present in a sample if ≥ 75% of the contig length was covered ≥ 1× by reads, as recommended (54, 80). Raw reads were normalized by vOTU genome length and library sequencing depth to generate counts per kilobase per million (CPM) using the following formula: [(raw reads/genome length)/sample read depth] × 1 $e^{6}$.

### Recovery of microbial populations

Microbial operational taxonomic units (OTUs) were recovered using bacterial and archaeal ribosomal protein S3 (rpS3) sequences, as previously described (41). Briefly, rpS3 sequences were identified by searching proteins predicted from the assembled metagenomes using a custom hidden Markov model. rpS3 protein taxonomy was subsequently inferred using BLASTp to search against a database of rpS3 proteins (81) with an *E*-value threshold of 1 $e^{-10}$. While the vast majority of OTUs were assigned to bacterial phyla, some OTUs were assigned to the archaeal phylum *Euryarchaeota* or unknown phyla.

In addition to OTUs, previously reconstructed (41) bacterial and archaeal metagenome-assembled genome sequences were accessed. Similarly, most of these genomes belonged to bacterial phyla.

To provide an alternative estimation of the taxonomic composition of bacterial communities, Kraken 2 v2.1.3 (82) was used to classify the unassembled metagenome reads. The composition of reads at the bacterial class level were recorded and used in analyses.

### Abundance of microbial populations and metagenome-assembled genomes

The abundance of OTUs and MAGs was estimated by mapping raw metagenome reads against rpS3-containing scaffolds and MAG genomes, respectively, using BBMap with a minimum alignment identity of 98%. OTUs and MAGs were only considered present in a sample if ≥ 75% of the contig length was covered. Coverage per base pair was normalized for sample sequencing depth using the following formula: (raw coverage/sample read depth) × average read depth across samples.

### Viral microdiversity

The nucleotide diversity (π) of viral populations and the proportion of non-synonymous to synonymous polymorphism ratio (pN/pS) of each viral gene in each sample were estimated with Metapop (83) using binary alignment map (BAM) files from read mapping (see above) and default parameters, including thresholds of > 70% genome coverage and > 10 × average read depth. The total microdiversity of each sample was calculated by averaging over bootstrapped π values, as previously described (84).

Genes under positive selection were identified with pN/pS ratios > 1. Genes encoding putative ABC transporters were further interrogated for the detection of conserved functional domains using CD-Search.

Consensus vOTU sequences were constructed using the most common allele from variant sites identified using inStrain v1.5.7 (85) and BAM files from read mapping. Variants were called if a site had a minimum of five viral scaffold reads. Strain-level heterogeneity was subsequently estimated by computing the pairwise average nucleotide identity (ANI) of these sample-specific consensus sequences. Pairwise comparisons were only considered for analysis when the genome coverage between samples was > 25%.

### Identification of anti-phage systems

Anti-phage systems were identified from MAGs using DefenseFinder (86, 87) (accessed May 2022), with default settings. Only MAGs carrying complete anti-phage systems, i.e., with all genes relating to the anti-phage system detected on the scaffold, were considered.

### Data analysis and visualization

**Fig 1:**
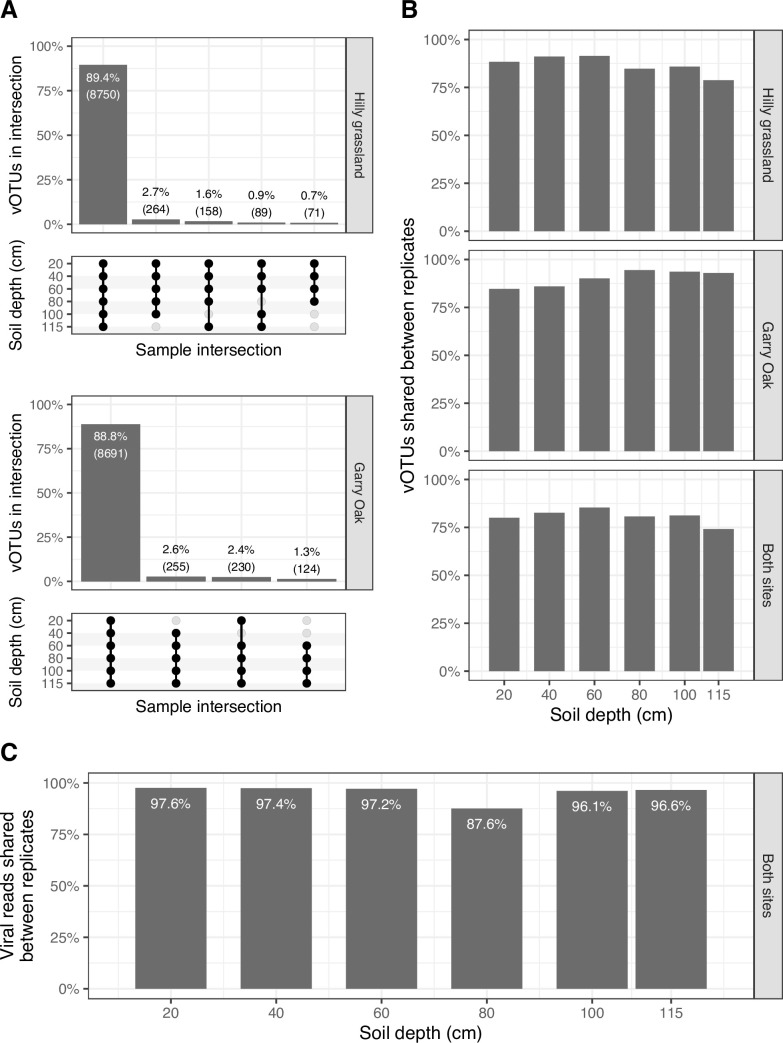
Distribution of viral populations throughout soil depth profiles. (A) Overlap in the detection of viral populations between different depths of each site. Intersection matrix denotes depths at which vOTUs were detected. Bar plot displays the percentage of total vOTUs detected in depth intersection. Bars shown for depth intersections containing more than 60 vOTUs. (B) Overlap in the detection of viral populations between the same depths (i.e., between replicates) of each site and both sites combined. (C) Percentage of viral reads shared between the same depths (i.e., between replicates) of both sites combined.

All statistical analyses were conducted using R v4.1.3 (59). Viral community alpha (within-sample) diversity was described with Simpson’s D index computed on vOTU CPM profiles with phyloseq v1.38.0 (88). Viral community evenness and dominance were estimated with Pielou’s J index and Berger-Parker’s D index, respectively. Viral community beta (between-sample) diversity was described by computing a Bray-Curtis dissimilarity matrix from square root transformed vOTU CPM values and subsequently visualized with non-metric multidimensional scaling (NMDS) ordination using vegan v2.6.2 (89). The same method was used for microbial community beta diversity, using normalized OTU coverage values. Permutational multivariate analysis of variance (PERMANOVA) tests and Mantel tests were also performed with vegan. Pearson’s correlation coefficients and linear regression slopes were calculated with stats v4.2.1. *P*-values were corrected for multiple testing when appropriate using the Holm algorithm. Differential abundance analysis was performed on raw read counts with DESeq2 v1.34.0 (90). Genome maps in Fig. S8 were visualized with gggenes v0.4.1 (91). Figure 1A and 3B; Fig. S4A was made with ComplexUpset v1.3.3 (92, 93). All remaining plots were generated with ggplot2 v3.3.6 (94).

## RESULTS

### Soil viruses were highly prevalent throughout the soil depth profiles

To investigate changes in viral communities with soil depth, we leveraged a publicly available metagenomic data set sampled from grassland soil in Northern California (41). Soil samples were previously collected in duplicate at six intervals between 20 cm and 115 cm below the soil surface, at two sites representing contrasting aboveground vegetation: under a Garry oak tree (“Garry Oak” samples) and neighboring grassland (“Hilly grassland” samples). In total, 24 assembled metagenomes were used to recover viral populations (vOTUs) using a combination of viral prediction tools. The quality of library assemblies is described in Table S1. This yielded 10,196 non-redundant vOTUs (> 5 kb), representing 9,664 dsDNA viral species and 532 ssDNA viral species (Table S2), with 292 vOTUs (2.9% of total) identified as complete or high-quality viral genomes. The mean vOTU genome length was ~12 kb, while 19 vOTUs had genome lengths > 200 kb (largest 415,894 bp) and represented “jumbo phages” (95), of which 18 were classified as high-quality viral genomes.

To estimate the similarity of recovered vOTUs with all currently available bacteriophage (phage) genomes (57), shared protein-based classification was performed using vConTACT2 (58) (Fig. S1). The resultant network contained viral clusters representing roughly genus-level taxonomic groups (Fig. S1A). There were 4,124 (42.7% of total) dsDNA vOTUs and 129 (24.2% of total) ssDNA vOTUs which formed 1,310 VCs and 89 VCs, respectively (Table S2). However, only 10 VCs included both our vOTUs and phage genomes that had been previously isolated, demonstrating the novel viral taxonomic diversity accessed from subsurface soil in this study. The analysis was expanded to include > 600,000 previously identified environmental viral sequences, using PhageClouds (61). Our vOTUs had intergenomic distances < 0.21 with only 85 previously discovered viral sequences in public databases (Table S3). Of the 75 viral sequences with available metadata at the time of analysis, 74 were assembled from soil.

While only one VC contained multiple jumbo phage vOTUs (cluster 259; three vOTUs), 63 vOTUs < 200 kb shared VCs with jumbo phage vOTUs (hereafter referred to as “jumbo-related” vOTUs). To investigate the diversity of these vOTUs further, we constructed a phylogeny of 24 DNA polymerase genes identified within the genomes of eight jumbo phage vOTUs and six jumbo-related vOTUs (Fig. S2). This revealed that the vOTUs belonged to six distinct phylogenetic groups, which we denoted A–F. Further investigation of the groups with the closest known relatives (groups A, B, and F) identified that the most similar DNA polymerase genes were carried by genomes < 200 kb, therefore representing non-jumbo phages (Fig. S3).

To gain an overview of the viral communities, we interrogated the distribution of vOTUs detected throughout the two soil depth profiles (Fig. 1). Of the 10,196 total vOTUs recovered, 9,783 (99.9%) of the 9,789 detected vOTUs were present in both sites, while 8,750 (89.4%) and 8,691 (88.8%) of vOTUs were detected across all depths in Hilly grassland and Garry Oak, respectively (Fig. 1A). This identified that viral prevalence was high throughout the two soil depth profiles. Moreover, a minimum of 78% of vOTUs was shared between the duplicate depth samples of each site, and a minimum of 74% of vOTUs was shared across all depth replicates across both sites (Fig. 1B). This resulted in a minimum of 87.6% of viral reads mapping to vOTUs shared across all depth replicates across both sites (Fig. 1C). A markedly different distribution was observed for microbial communities, which displayed a narrower distribution throughout the depth profiles and a lower similarity between replicates, particularly between sites (Fig. S4).

### Subsurface soil viral communities were increasingly dissimilar between sites

**Fig 2:**
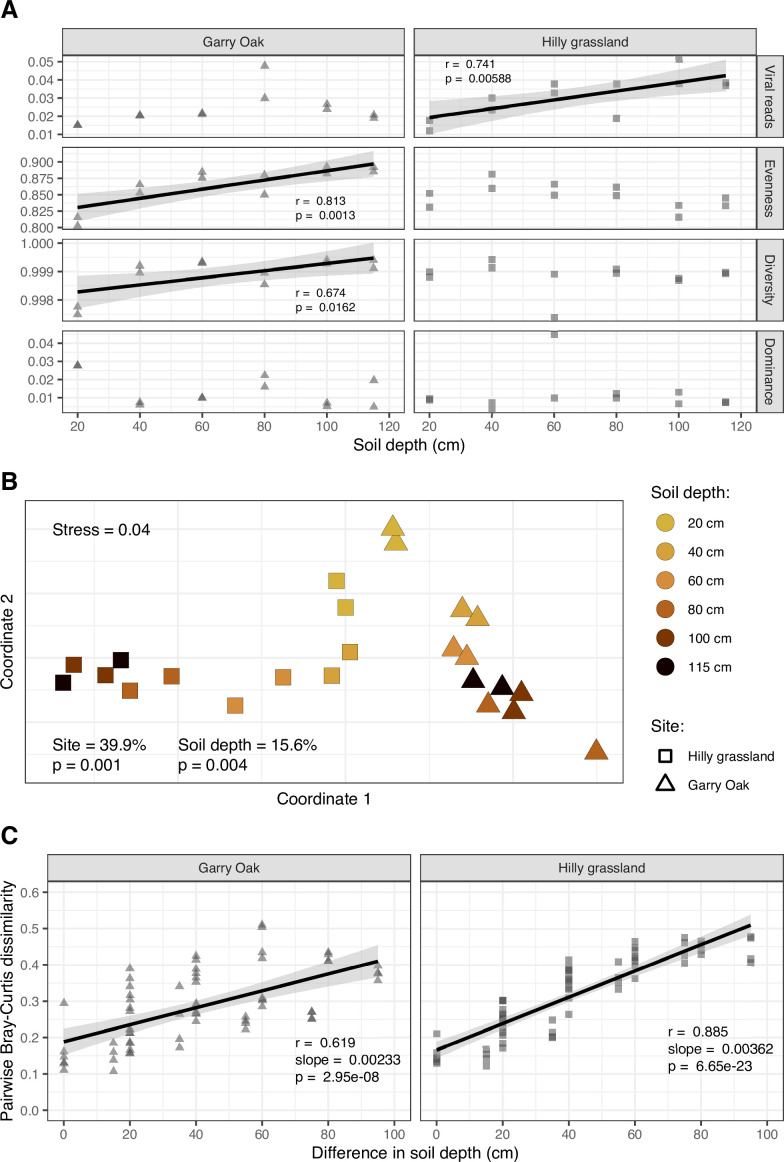
Population-level assembly of soil viral communities throughout soil depth. (A) Alpha diversity of viral communities. Viral reads (as a proportion of total raw reads), Evenness (Pielou’s *J* index), alpha diversity (Simpson’s *D* index), and dominance (Berger-Parker’s *D* index) for each viral community throughout the soil depth profiles. Trend lines represent linear regression estimates, with shaded cloud representing 95% CI. r corresponds to Pearson’s correlation coefficient and p corresponds to the associated *P*-value. (B) Beta diversity of viral communities. Non-metric multidimensional scaling ordination plots, representing the Bray-Curtis dissimilarities between viral community compositions. Shapes indicate site: Garry Oak (triangles) and Hilly grassland (squares). Shapes are colored based on soil depth. Stress value associated with two-dimensional ordination is reported. Percentage contribution to variance by site and soil depth, as calculated with a permutational multivariate analysis of variance test, and associated *P-*value are also reported. (C) Distance-decay relationship in viral community structure. Trend lines represent linear regression estimates, with shaded cloud representing 95% CI. r corresponds to Pearson’s correlation coefficient, slope corresponds to linear regression slope, and p corresponds to the associated *P*-value.

Given the high prevalence of vOTUs and the similarity in their detection between the two depth profiles, we next sought to characterize the role of soil depth in shaping the structure of viral communities by assessing population-level viral diversity throughout soil depth (Fig. 2). Significant correlations were identified for viral evenness (measured with Pielou’s J index) and viral diversity (measured with Simpson’s D index) with soil depth in Garry Oak only (Fig. 2A). It was confirmed that viral diversity was not an artefact of sequencing depth or the proportion of total viral reads per sample library, given their non-significant associations. However, the proportion of total raw reads that were captured by vOTUs did increase with soil depth in Garry Oak (Fig. 2A). No depth relationship was observed for community dominance (measured with Berger-Parker’s D index) in either site (Fig. 2A).

Next, we tested whether soil depth was an ecological driver of viral community composition through NMDS ordination and a PERMANOVA test. Bray-Curtis dissimilarities were correlated with soil depth ($R^{2}=$ 0.156, F= 7.37, *P* = 0.002) (Fig. 2B), such that significant distance-decay relationships were observed at both sites (Fig. 2C). Additionally, viral communities were distinct between sites, with aboveground vegetation explaining more than twice the variation as soil depth ($R^{2}=$ 0.399, F= 18.8, *P =* 0.001) (Fig. 2B).

**Fig 3:**
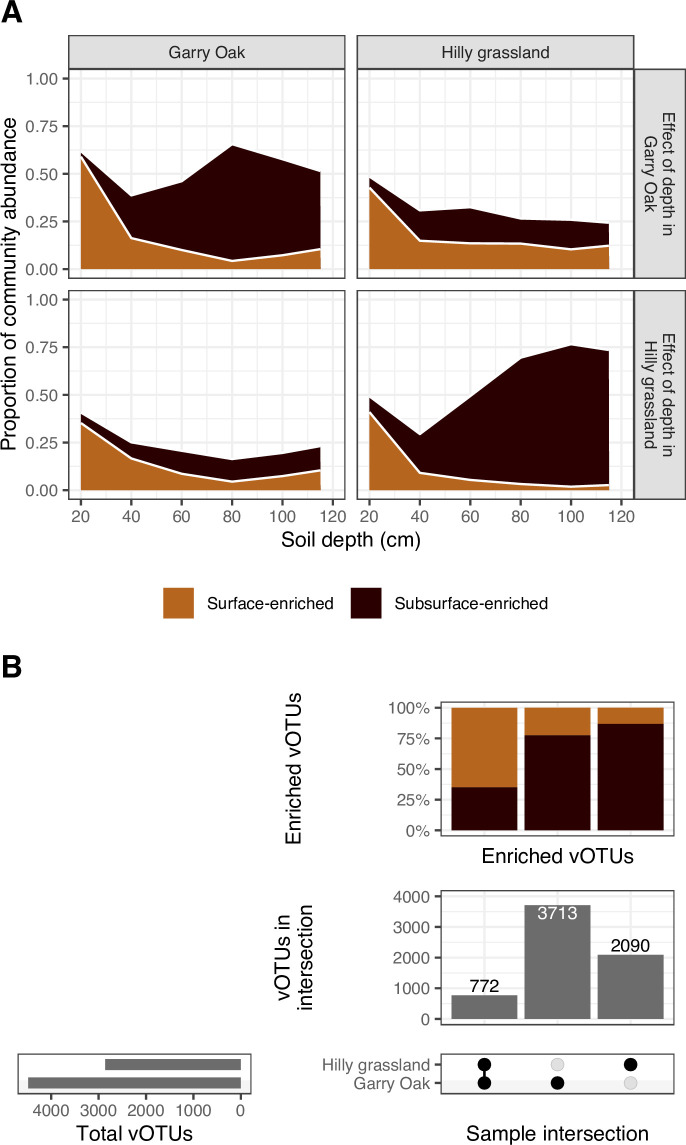
Overlap in depth enrichment of viral populations between sites. (A) Relative abundance of depth-enriched viral populations. Proportional abundance of vOTUs enriched in either surface soil (20 cm) or subsurface soil (40 cm–115 cm) based on samples derived from Garry Oak and Hilly grassland, across Garry Oak and Hilly grassland samples, respectively. Colors indicate enrichment: surface enriched (light-brown) or subsurface enriched (dark-brown). (B) Overlap in depth enrichment of viral populations between sites. Intersection matrix denotes site investigated (bottom-right). Horizontal bar plot displays the total vOTUs detected in each site (bottom-left). Vertical bar plots display the number of enriched vOTUs in site intersection (middle-right) and the percentage of enriched vOTUs corresponding to surface enriched or subsurface enriched, respectively (top-right).

To further contrast the soil depth patterns between sites, we assessed relative viral abundances to identify populations enriched in either surface or subsurface soil (Fig. 3). Despite the high viral prevalence noted previously (Fig. 1A), differential abundance analysis revealed that > 29% of vOTUs had enriched abundance in either surface soil (20 cm) or subsurface soil (40 cm–115 cm) (Table S2). In comparing the relative abundance of the enriched viral populations between the two sites, we found that the vOTUs highly abundant in subsurface soil in one site were consistently less abundant throughout the soil depth profile in the other site (Fig. 3A). Subsequently, only 11.7% of depth-enriched viral populations was enriched in both sites, with 64.9% of these populations being surface-enriched (Fig. 3B). In fact, subsurface-enriched viral populations in each site were genetically different, as the shared populations represented only 18.5% of subsurface-enriched VCs in Garry Oak (Fig. S1B) and 13.5% in Hilly grassland (Fig. S1C). Together, these results outline the increased distinction of subsurface soil viral communities between sites.

**Fig 4:**
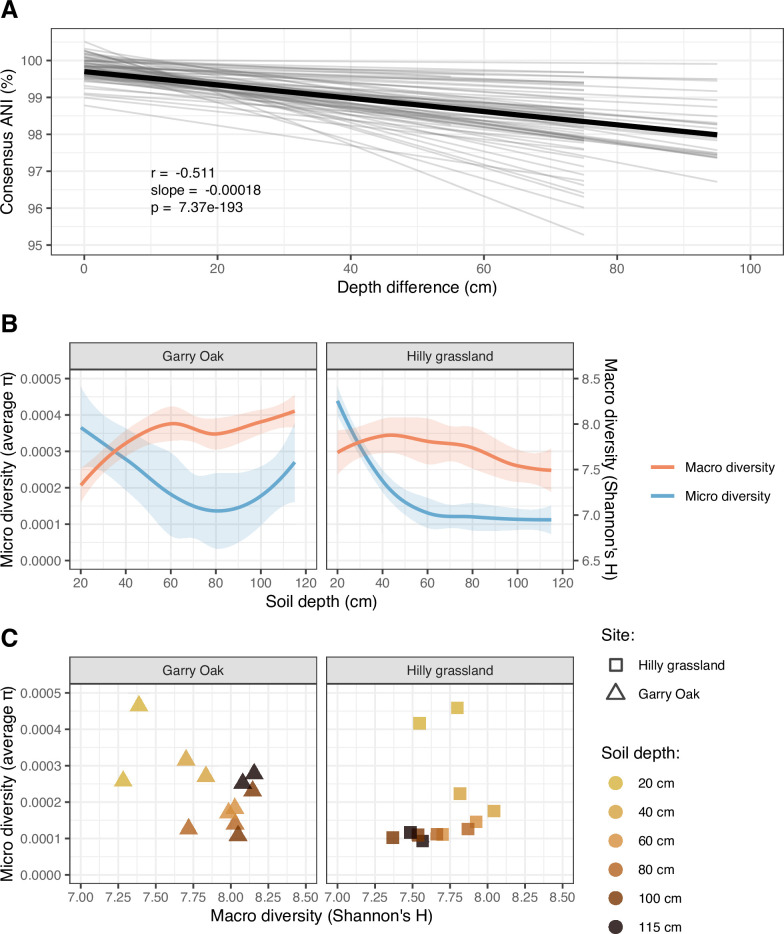
Strain-level assembly of soil viral communities throughout soil depth. (A) Distance decay relationship in consensus ANI. Lighter gray lines represent distance-decay relationships in consensus ANI for 69 vOTUs with individual significant relationships. Thicker black line represents the mean distance decay relationship across all 69 vOTUs. Trend lines represent linear regression estimates, with shaded cloud representing 95% CI. r corresponds to Pearson’s correlation coefficient, slope corresponds to linear regression slope, and p corresponds to the associated *P*-value. (B) Viral macrodiversity and microdiversity throughout the soil depth profiles. Trend lines represent loess smooth regression estimates, with shaded cloud representing 95% CI. Color indicates level of diversity: macrodiversity (red), microdiversity (blue). (C) Correlation of macrodiversity with microdiversity. Shapes indicate site Garry Oak (triangles) and Hilly grassland (squares). Shapes are colored based on soil depth.

Lastly, we investigated the effect of soil depth in driving patterns of strain-level viral diversity (Fig. 4). To achieve this, consensus sequences were reconstructed for each vOTU in each sample, based on the most common alleles detected across variant sites. Subsequent distance-decay relationships were observed across strains of 69 vOTUs, for which the pairwise ANI between consensus sequences decreased towards 0.95 (the threshold for vOTU clustering) with soil depth (Fig. 4A). To summarize the microdiversity across viral populations of each sample, the average nucleotide diversity (π) was assessed. This summarizes the frequency of nucleotide differences between the individual strains of a population. Average nucleotide diversity was greatest in surface soil and displayed a non-linear relationship with soil depth (Fig. 4B). As a result, no significant relationship was observed between population-level diversity (i.e., macrodiversity) and strain-level diversity (i.e., microdiversity) in either site (Fig. 4C).

### Virus-host interactions were diverse with soil depth

**Fig 5:**
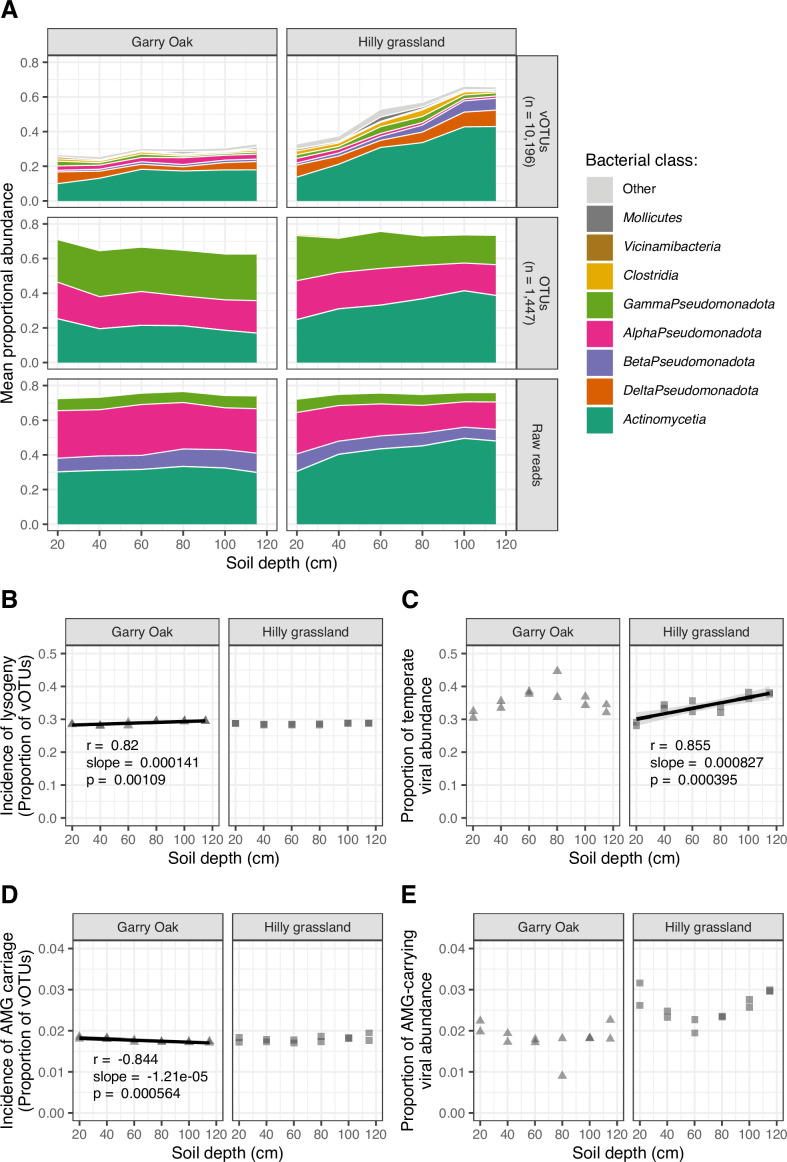
Virus-host interactions throughout soil depth. (A) Virus-host linkages. Mean proportional abundance is plotted across soil depth for vOTUs by predicted host class (*n* = 10,196), microbial OTUs by class (*n* = 1,447), and raw read taxonomy estimated by Kraken 2. Colors indicate bacterial class. (B) Incidence of lysogeny. Proportion of vOTUs detected representing temperate viruses plotted across soil depth. ( C) Temperate viral abundance. Proportional abundance of vOTUs detected representing temperate viruses plotted across soil depth. (D) Incidence of AMG carriage. Proportion of vOTUs carrying AMGs plotted across soil depth. (E) AMG-carrying viral abundance. Proportional abundance of vOTUs carrying AMGs plotted across soil depth. For (B–D), trend lines represent linear regression estimates, with shaded cloud representing 95% CI. r corresponds to Pearson’s correlation coefficient, slope corresponds to linear regression slope, and p corresponds to the associated *P*-value.

To explore the potential ecological roles of viruses with soil depth, we characterized the interactions between viruses and their microbial host communities (Fig. 5). Strong links were revealed between viruses and microbes by observing significant correlations between their community structures (Fig. S5A) and diversities (Fig. S5B). To provide further evidence of virus-host linkages, we identified the putative host taxa of vOTUs using a combination of proviral scaffold assessment, CRISPR spacer matches, and *de novo* prediction using a probabilistic model (75). This predicted hosts for 3,324 (32.6% of total) vOTUs, with *Actinomycetota* and *Pseudomonadota* being the most common host phyla (Table S2). Moreover, viruses infecting *Actinomycetia* were prominent members of viral communities throughout the soil depth profiles, particularly in Hilly grassland (Fig. 5A). While the composition of microbial classes described from OTU abundances and raw read taxonomy differed, both approaches demonstrated that the abundance of *Actinomycetia* increased with depth in Hilly grassland (Fig. 5A). Using host class abundances estimated from OTUs (Table S4), significant correlations were identified with viruses of *Actinomycetia* and *AlphaPseudomonadota* in Hilly grassland only (Fig. S6A). When using host class abundances estimated from raw read taxonomy (Table S5), significant correlations were identified with viruses of *Actinomycetia*, *AlphaPseudomonadota*, and *Clostridia* in Hilly grassland and *DeltaPseudomonadota* and *Vicinamibacteria* in Garry Oak (Fig. S6B).

Given that viral replication strategies inform virus-host interactions following infection, we investigated the prevalence of lysogeny with soil depth through the detection of temperate viruses. In total, 2,911 (28.6% of total) temperate viruses were detected. The incidence of lysogeny, as measured by the proportion of detected vOTUs which were identified as temperate, was stable throughout soil depth (Fig. 5B). In contrast, the relative abundance of temperate viruses varied, such that a positive relationship with soil depth was observed in Hilly grassland (Fig. 5C).

In addition to host cell lysis, another fundamental ecological role of viruses is the alteration of host metabolism through the expression of AMGs during infection. We identified 220 putative AMGs carried by 181 vOTUs (1.77% of total; Table S6), whose functional annotations included hits to ribosomal proteins (nine genes) and carbohydrate-active enzymes (CAZymes; 43 genes). Six jumbo phage vOTUs carried a single AMG each, while the average length of vOTUs carrying multiple AMGs was 29,600 bp. vOTUs carrying AMGs were consistently detected throughout the soil depth profiles, with a small yet statistically significant decrease in incidence with depth in Garry Oak (Fig. 5D). No significant depth relationships were observed for the relative abundance of AMG-carrying vOTUs (Fig. 5E).

Further inspection of candidate CAZymes with CD-Search revealed that 36/43 (83.7%) gene products contained conserved protein domains associated with carbohydrate metabolism (Table 1). This included 12 genes with glycoside hydrolase domains, putatively involved in the metabolism of four different carbon sources: glycans (five genes), amylose (two genes), cellulose (two genes), and mannose (one gene). vOTUs carrying CAZymes were dispersed across 21 VCs and 17 singletons in the shared protein network (Fig. S1D). Three-quarters of vOTUs carrying CAZymes were lytic and 17/40 (42.5%) had predicted hosts, with *Actinomycetia* being the most common host class (47% of vOTUs with predicted hosts). The vOTUs were detected throughout the two soil depth profiles, at consistently low abundance (Fig. S7).

**TABLE 1:** Summary of carbohydrate-active enzyme identification

Enzyme class	Class function	Most common enzyme family (number of genes)	Number of viral genes		
			Total	With conserved domains	On high-quality viral genomes
Glycosyl transferase	Catalyzes the transfer of sugar moieties to form glycosidic bonds	GT4 (6)	17	15	2
Glycoside hydrolase	Catalyzes the hydrolysis of glycosidic bonds	GH33 (3)	13	12	1
Carbohydrate esterase	Catalyzes the de-acetylation of substituted saccharides	CE4 (4)	7	7	0
Carbohydrate-binding molecule	Non-catalytic proteins appended to carbohydrate-active enzymes	CBM66 (4)	6	2	1

### Virus-host antagonistic co-evolution was dynamic throughout the soil depth profile

**Fig 6:**
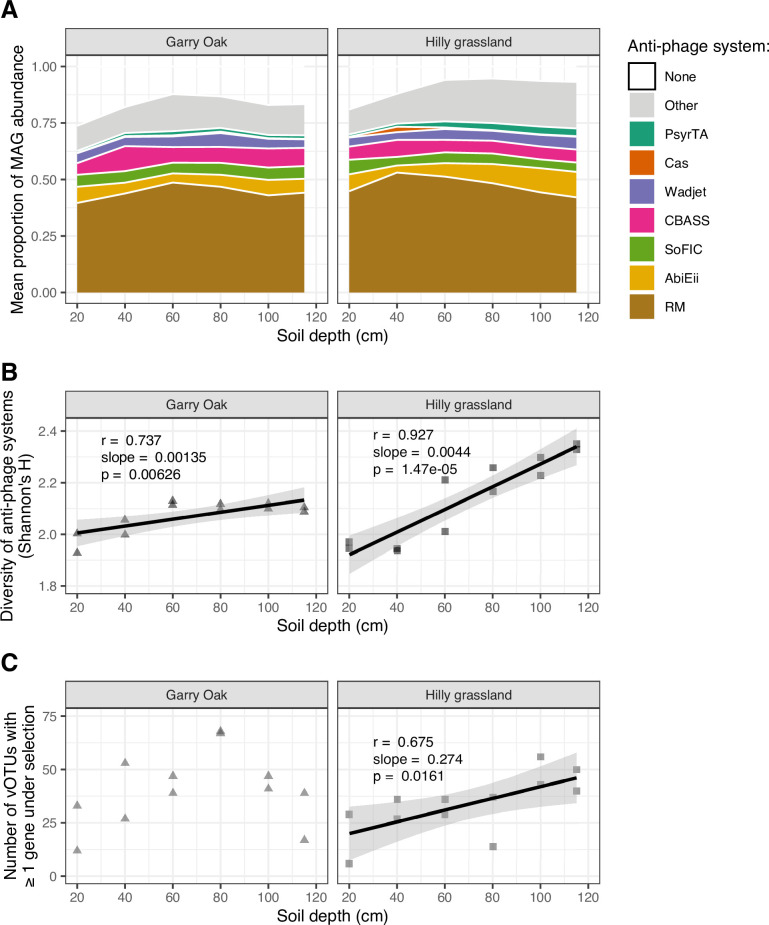
Virus-host antagonistic co-evolution throughout soil depth. (A) Anti-phage system detection. Proportional abundance of microbial MAGs carrying complete anti-phage systems. Color indicate anti-phage system. (B) Diversity of the anti-phage system repertoire. Shannon’s *H* index, calculated on MAGs carrying complete anti-phage systems, plotted across soil depth. (C) Viruses under positive selection. Number of vOTU genomes with at least one gene under positive selection (indicated by a pN/pS ratio > 1) plotted across soil depth. For (B and C), trend lines represent linear regression estimates, with shaded cloud representing 95% CI. r corresponds to Pearson’s correlation coefficient, slope corresponds to linear regression slope, and p corresponds to the associated *P*-value.

Virus-host interactions can also have implications on the eco-evolutionary dynamics of both viruses and microbes. Thus, to investigate virus-host antagonistic co-evolution throughout the soil depth profile, we identified bacterial anti-phage defense systems and estimated the subsequent selection pressure applied to soil viruses (Fig. 6). More than 75% of microbial community abundance was represented by MAGs carrying at least one complete anti-phage system, with systems involving restriction-modification (RM) being the most common (Fig. 6A; Table S7). Further investigation into the anti-phage system repertoire of MAG communities revealed a significant increase in system diversity with soil depth in both sites (Fig. 6B).

To assess the resulting evolutionary pressures on viral populations, we identified viral genes under positive selection using a proportion of non-synonymous to synonymous polymorphism ratio (pN/pS) > 1. This yielded 532 vOTUs carrying 880 genes under positive selection in at least one sample, with nearly half of these genes lacking functional annotations (Table S8). Nonetheless, we were able to identify functions for 30 tail fiber proteins involved in host cell recognition (96, 97), four tape measure proteins involved in virion assembly (98) and genome insertion (99), six ribosomal proteins, and 11 ABC transporters (Table S8). Manual inspection of putative ABC transporter genes with CD-Search indicated the presence of conserved secondary structures for 10 of the genes, with five genes containing drug efflux transporter domains (*ccmA*, *drrA*, *MacAB*, *MacB*, and *SunT*). Moreover, five vOTUs carrying ABC transporter genes represented high-quality temperate viral genomes, with hits to viral protein families (i.e., PHROGs) both upstream and downstream of putative transporter genes (Fig. S8). While only one ABC transporter gene was positively selected in surface soil (20 cm), the remaining 10 genes were positively selected in subsurface soil (40 cm–115 cm). Overall, the number of vOTUs carrying at least one gene under positive selection increased with soil depth in Hilly grassland, while no linear relationship was observed with soil depth in Garry Oak (Fig. 6C).

## DISCUSSION

### Virus-host co-existence was high throughout soil depth

Microbial dispersal underpins soil ecology and evolution (100); however, we lack the understanding of the vertical distribution patterns of soil viruses. In this study, we observed high viral prevalence throughout two soil depth profiles, with > 88% of viral populations (vOTUs) detected at every sampled depth (Fig. 1A). This cosmopolitan distribution contrasted with recent investigations of soil viral dispersal, in which fewer viruses were shared between samples across horizontal (14, 16, 17, 101, 102) and vertical spaces (18, 101, 103). Despite high viral prevalence, we discovered that soil depth shaped the assembly of viral communities (Fig. 2B), such that viral community diversity displayed a distance-decay relationship (Fig. 2C).

The structuring of viral communities with soil depth is undoubtedly dependent on the physical structure of the soil matrix, which renders virion dispersal a mostly stochastic process (100). The rate-limiting factors underlying the transport of viruses through soil are likely different to those of their hosts (32, 104). Notably, soil viruses are expected to be passively distributed with water more easily (105). Therefore, wetter soils may facilitate the enhanced mobility of viruses compared with their hosts, resulting in the increased accessibility and infection of susceptible host cells. This could explain the disparity in the distribution patterns observed here between viruses (Fig. 1) and microbes (Fig. S4), albeit across two replicates within in each site. Simultaneously, the abundance of viruses has been correlated with soil moisture content (14, 16, 17, 101), demonstrating how environmental factors may affect virus-host interactions.

At the Sagehorn site in California where soil samples were taken, significant winter precipitation raises the water table close to the soil surface (106). The resulting annual saturation of soil may facilitate the immigration of infective viruses and susceptible hosts throughout the soil depth profile. This would have consequences on both bacteriophage (phage) and bacterial persistence due to evolutionary “source-sink dynamics,” where co-existence is maintained by the heterogeneous distribution of viruses and hosts (107, 108). This has been demonstrated in biofilm simulations, whereby the mobility of virions is a key determinant of phage-bacteria co-existence (109). Therefore, we propose that the high viral dispersal is likely to have implications on the eco-evolutionary interactions occurring across the soil niches examined in this study.

### Soil depth patterns of viral community composition were different between sites

Unfortunately, the present study design was limited in its investigation of only two depth replicates within two sites. Nonetheless, we observed that the variation in viral communities between sites was greater than the variation associated with soil depth (Fig. 2B), such that communities in subsurface soils were more dissimilar than those at the surface (Fig. 3). A considerable distinction between the two sites investigated here was the presence of Garry Oak trees, for which the annual shedding of leaves during winter has been reported (47). Decaying leaf litter has been shown to shape the composition of RNA viral communities in both the rhizosphere and bulk soil (110). While quicker degradation rates mean that the spatial structuring of RNA viruses may be greater than for DNA viruses, the legacy effects of leaf litter may have driven differences between surface soils. However, the degradation of shed leaves would be expected to have a weaker effect on subsurface communities. Instead, we hypothesize that the presence of tree roots and associated fungal hyphae at the study site (47) impacted viral communities in Garry Oak samples, leading to the discrepancies in the depth patterns between the two sites. The effects of roots and hyphae on bacterial diversity and activity could indirectly impact viral communities through the changes to their host community. Indeed, the influence of growing crop roots on the structures of both DNA and RNA soil viral communities has been reported previously, where viral community structure and activity were distinct in the surface rhizosphere (13). While we propose this hypothesis to be driving site differences, we acknowledge that this was based on a small study with only two sites. In the future, larger scale studies with multiple independent sites would be required to test this hypothesis.

### Temperate viruses were not dominant in subsurface soil

Lysogenic viral infections can have significant eco-evolutionary impacts on host communities (30), most notably through superinfection exclusion, which confers resistance against further viral infection (111–113). Typically, lysogeny is expected to dominate in soil ecosystems because of low host biomass and viability (28, 29, 35). Under low bacterial densities (e.g., < 10^5^ cells per gram of soil), host starvation represses viral lytic genes through ATP-dependent signaling cascades (114, 115), promoting lysogeny switching (116). Subsequently, lower bacterial abundances have been associated with increased lysogeny in the deep ocean (117–119). Recent work has observed an increased prevalence of lysogeny in subsurface soils, as detected through inducible lysogens (28); however, here, we observed very little change in the incidence of temperate viruses (Fig. 5B). And while the relative abundance of temperate viruses did increase with soil depth in Hilly grassland, this was not consistent in Garry Oak (Fig. 5C). Therefore, there could be additional factors which govern the selection of temperate viruses in soils beyond host density. This could include non-linear relationships with host metabolism (116), viral-viral interactions (120, 121), and anti-phage defense systems (87). To this point, the diversity of anti-phage defense systems was enriched among subsurface communities in Hilly grassland (Fig. 6B), coinciding with the increased abundance of temperate viruses. The increased encountering of lysogenic infection mechanisms may have been responsible for the greater range of defense systems maintained among the host community (87). It must also be noted that viruses without lysogenic genes can establish passive co-existence typified by temperate lifestyles, as demonstrated with ΦcrAss001 in continuous culture with its host *Bacteroides intestinalis* (122). Therefore, non-lysogenic phages may be able to replicate without eradicating their host population, in contrast to the traditional view of predator-prey cycles induced by lytic phages.

### Jumbo phages recovered from soil were polyphyletic

We recovered 19 vOTUs representing jumbo phages (95) with genome lengths > 200 kb (largest 415,894 bp), without implementing a viral contig binning approach. An additional 63 vOTUs formed roughly genus-level VCs with jumbo phages, and together, they represented six distinct clades based on DNA polymerase gene phylogeny (Fig. S2). This is consistent with previous findings that jumbo phages are polyphyletic, implying that phage genome gigantism has evolved numerous times instead of originating from a single common ancestor (123, 124). Furthermore, the phylogeny revealed that the closest known relatives to jumbo phage vOTUs had much shorter genomes (Fig. S3). It has been postulated that jumbo phages may have evolved from recombination events between multiple smaller phage genomes (123). Another potential hypothesis for the origin of phage genome gigantism is that the genomes could have expanded upon the acquisition of additional phage or host genes. The ratchet model describes how mutations that increase the capsid size facilitate the acquisition of new viral genes, which are then stable against loss of function mutations (125).

Previously identified clades of jumbo phages have been discerned by their diverse infection and replication strategies, biogeography, and host taxa (123, 124). Here, we report a ubiquity of jumbo phages across two soil depth profiles, suggesting that large genome sizes are evolutionarily stable across both surface and subsurface soil niches. Furthermore, jumbo phages were consistently in the top 20% of the most abundant viruses in each community (Fig. S9A), contrasting with previous findings that giant viruses (> 300 kb) are less abundant in forest soil (126).

### Soil viruses have the potential to augment microbial metabolism in both surface and subsurface soils

Viruses can carry and express AMGs during infection to modulate the host’s metabolism and fitness and promote their co-existence (2–6). Moreover, viral-encoded AMGs have the potential to affect soil biogeochemistry, with viruses previously implicated in soil carbon processing (13, 18, 19, 34, 101, 127). In this study, we detected viruses throughout both soil depth profiles carrying CAZymes associated with both carbohydrate anabolism and catabolism (Table S6). The rank abundance of CAZyme-carrying viruses was highly variable, but their presence was ubiquitous across all soil depths (Fig. S9B). Therefore, soil viruses may stimulate the degradation of a variety of carbon sources, including plant cell walls, thus contributing to the remineralization of soil carbon in surface and subsurface soils. While our discovery of viral CAZymes adds to the repertoire of potential viral mechanisms contributing to soil carbon cycling, evidence of their function during the infection cycle has not been confirmed here.

Previously, the abundance of viral-encoded AMGs was found to increase with soil depth (103). However, we observed that the abundance of viruses carrying AMGs was consistently low throughout both soil depth profiles (Fig. 5D and E). The most common host class of viruses carrying AMGs was *Actinomycetia*, for which both the host (Fig. 5A) and infecting viruses (Fig. S10) were more abundant in subsurface soil. *Actinomycetia* (formerly *Actinobacteria*) are dominant soil microbes (128) and contribute to soil carbon cycling by producing extracellular hydrolytic enzymes which depolymerize plant-derived lignin (129). Furthermore, *Actinomycetia* are resilient to soil drying, such that their relative abundance increases during drought and declines in the days following re-wetting (130–132). The abundance and activity blooms in response to seasonal wetting and drying are likely to affect soil nutrient and carbon cycling (132).

### Viral macrodiversity and microdiversity were associated with surface soil only

The evolution of viral communities can be monitored through microdiversity. In this study, we have revealed patterns of viral microdiversity throughout two soil depth profiles. In doing so, we demonstrated that viral strain-level heterogeneity displayed a distance-decay relationship (Fig. 4A) and the average microdiversity (π) of viral communities varied across space (Fig. 4B).

Microdiversity is accrued through *de novo* mutations and can drive phenotypic variation to specialize organisms to their environment (85). More specifically for viruses, microdiversity reflects evolutionary responses to host infection dynamics and is directly related to viral infection rates. Greater viral microdiversity, as measured by larger π values, can arise in multiple ways (83). First, the active infection of hosts can result in population expansion and thus more frequent mutations. This can be exacerbated through genetic recombination between viral populations co-infecting the same host. Such horizontal gene transfer events are made more likely by the presence of microbial “hotspots” occurring throughout the spatially structured soil matrix (133). Second, viral populations could maintain greater microdiversity in their populations as an evolutionary mechanism. Genetic diversity increases the fitness of a viral population by allowing them to “bet-hedge” if their environment or host changes, conferring local adaptation (134).

The ecological forces driving strain-level variation were apparently distinct from those driving population-level variation, as demonstrated by their non-significant association (Fig. 4C). This was surprising given that genetic heterogeneity between strains can result in speciation events (134, 135), thus relating the two levels of diversity. Throughout ocean depth profiles, a similar absent relationship was explained by interactions with bacterial macrodiversity (84). However, no such relationship was observed in these soil samples. Instead, we propose three mechanisms by which this relationship may have been obscured. First, due to the metagenomic approach taken in this study, there was no way to estimate viral activity, and so, it is possible that the viral communities described here included inactive viruses. While the detection of inactive viruses would have contributed to the estimated population-level variation, they would have had minimal contribution to the strain-level variation, expected to have been accrued through active infection and replication. Subsequently, the disproportionate contribution of inactive viruses to macrodiversity may be confounding its association with microdiversity. Second, metagenomes lacking the enrichment of viral particles have been shown to access viral diversity less effectively, as compared with enriched metagenomes (i.e., DNA viromes) (16). This may have limited our ability to access the true viral macrodiversity and microdiversity from the microbial-dominated metagenomic libraries. Third, we speculate that unmeasured physicochemical properties, distinct between soil horizons, may have contributed to the non-linear diversity dynamics observed throughout the soil depth profile.

Nonetheless, when the analysis of viral diversity patterns was focused on the top 60 cm of soil, viral macrodiversity was found to be negatively associated with viral microdiversity (Fig. 4B). This could have resulted from decreasing host cell density from surface to subsurface soil (26), which favors inter-specific viral competition (i.e., reflected in macrodiversity) over intra-specific viral competition (i.e., reflected in microdiversity). Hence, strain-level heterogeneity is less favored when fewer hosts are available, during which species-level competition drives evolution. This would be expected to impact virus-host interactions by reducing the resilience of the subsurface soil niche.

### Antagonistic co-evolution was distinct among surface and subsurface communities

Host defense responses to viral infection are expected to drive positive selection among soil viruses through antagonistic co-evolution. To this aim, we identified 880 viral genes under positive selection (Table S8), for which non-synonymous polymorphisms were more likely to be retained than rejected. This included 30 tail fiber genes, which have previously been shown to be positively selected among gut phages as evidence of their adaptive evolution (136, 137). Phage tail fiber proteins are involved in host tropism (96, 97); thus, the carriage of genetically diverse tail fiber genes may expand a population’s host range. Given the positive selection of tail fiber gene mutants throughout the two soil depth profiles, the evolutionary benefit of expanding the host range was universal among viruses occupying both surface and subsurface soil niches. More generally, this could implicate broad host ranges as an adaptive feature of soil viruses.

We also identified 11 ABC transporter genes under positive selection, predominantly in subsurface soil (40 cm–115 cm) (Table S8). Five vOTUs carrying ABC transporter genes represented high-quality temperate viral genomes (Fig. S8), with two of these genes sharing conserved protein domains with ABC drug efflux transporters. By expressing these genes during infection, temperate soil viruses may confer antibiotic resistance to their hosts, thus maintaining their mutual co-existence. Furthermore, evidence of adaptive evolution among these genes indicates that there is a selection pressure on these viruses to augment their host’s interbacterial competition. While this may be the first evidence of soil viruses carrying ABC transporters, the expression of phosphate-binding *pstS* genes by cyanophages has implicated marine viruses in enhancing phosphate uptake in cyanobacterial hosts (138). Many other viral genes under positive selection had no functional annotation, suggesting that we may be missing additional selection pressures on soil viruses. For example, missing annotations may include uncharacterized anti-defense proteins, expressed by viruses to target host defense systems and maintain infective capabilities (139).

To characterize the range of host defense responses to viral infection, we identified anti-phage defense systems within microbial MAGs. The relative abundance of MAGs adopting at least one system was high throughout the soil depth profile (Fig. 6A), and the increasing diversity of anti-phage systems (Fig. 6B) suggested that the antagonistic co-evolution landscape differed between surface and subsurface niches. Multiple anti-phage defense systems can be carried within defense islands (140), a genetic toolbox of diverse mechanisms to resist viral infection, presumably accrued through horizontal gene transfer events (139). The genetic diversity of infecting viruses can direct the evolution of host defense strategies, such that low viral diversity may favor CRISPR-based immunity, while higher viral diversity promotes surface modification mechanisms (141). Thus, the microheterogeneity driven by the soil matrix would make these virus-host interactions difficult to predict.

### Conclusions

Most soil viral ecology efforts have focused on the top 20 cm of soil, hindering our understanding of subsurface viruses. Given the exponential decay in microbial biomass with soil depth, one might expect relatively minimal ecological impacts of subsurface viral communities. To the contrary, we have uncovered evidence for the potential of soil viruses to contribute to terrestrial ecology in both surface and subsurface soil niches. The prevalence of lysogeny was consistent throughout the soil depth profiles, indicating that additional factors beyond host cell density may govern the selection of temperate phages in soils. By investigating viral microdiversity patterns across the soil depth gradient, we revealed that the local adaptation of viruses was greatest in surface soil. Furthermore, an increasing diversity of anti-phage defense systems with depth suggests that the antagonistic co-evolution landscape may be distinct in subsurface soil. In the future, we predict that comparative activity studies, contrasting surface and subsurface niches, will be essential to characterize viral functions associated with soil depth.

## Data Availability

The metagenomic data set can be accessed from NCBI under project accession PRJNA577476 (sample accessions SAMN13153360-SAMN13153383). DNA vOTU genome sequences were deposited to the European Nucleotide Archive (ENA) under project accession PRJEB57765 (sample accession SAMEA112154074). FASTA nucleotide files containing vOTU genomes, FASTA amino acid files containing vOTU genes, vOTU gene annotations, vConTACT2 network input and output files, rpS3 protein sequences, and assembled MAG sequences are available from figshare (https://doi.org/10.25392/leicester.data.21647672). The custom R script used to generate figures and tables, along with required data files, is available from GitHub (https://github.com/GeorgeMuscatt/GrasslandDepthVirome).

## SUPPLEMENTAL MATERIAL

Fig. S1 mbio.02246-23-s0001.pdf Taxonomic novelty of recovered soil vOTUs.

Fig. S2 mbio.02246-23-s0002.pdf Phylogenetic assessment of jumbo phage vOTUs and jumbo-related vOTUs using DNA polymerase gene.

Fig. S3 mbio.02246-23-s0003.pdf Phylogenetic groups A, B, and F from assessment of jumbo phage vOTUs and jumbo-related vOTUs using DNA polymerase gene.

Fig. S4 mbio.02246-23-s0004.pdf Distribution of microbial populations throughout soil depth profiles.

Fig. S5 mbio.02246-23-s0005.pdf Correlation of viral community and microbial community.

Fig. S6 mbio.02246-23-s0006.pdf Correlation of viral abundances and host abundances.

Fig. S7 mbio.02246-23-s0007.pdf Relative abundance of viruses carrying carbohydrate-active enzymes.

Fig. S8 mbio.02246-23-s0008.pdf Genome maps of high-quality viral genomes carrying ABC transporters under positive selection.

Fig. S9 mbio.02246-23-s0009.pdf Rank abudance of jumbo phages and viruses carrying CAZymes.

Fig. S10 mbio.02246-23-s0010.pdf Relative abundance of viruses carrying auxiliary metabolic genes by host class.

ASM does not own the copyrights to Supplemental Material that may be linked to, or accessed through, an article. The authors have granted ASM a non-exclusive, world-wide license to publish the Supplemental Material files. Please contact the corresponding author directly for reuse.
