# Supplementary material for: Viral metagenomics reveals diverse virus-host interactions throughout the soil depth profile
Source: mBio. 2023 Nov 30;14(6):e02246-23. doi: 10.1128/mbio.02246-23 (PMC10746233; doi:10.1128/mbio.02246-23)
Supplement: Fig. S1 — Taxonomic novelty of recovered soil vOTUs. [file mbio.02246-23-s0001.pdf]

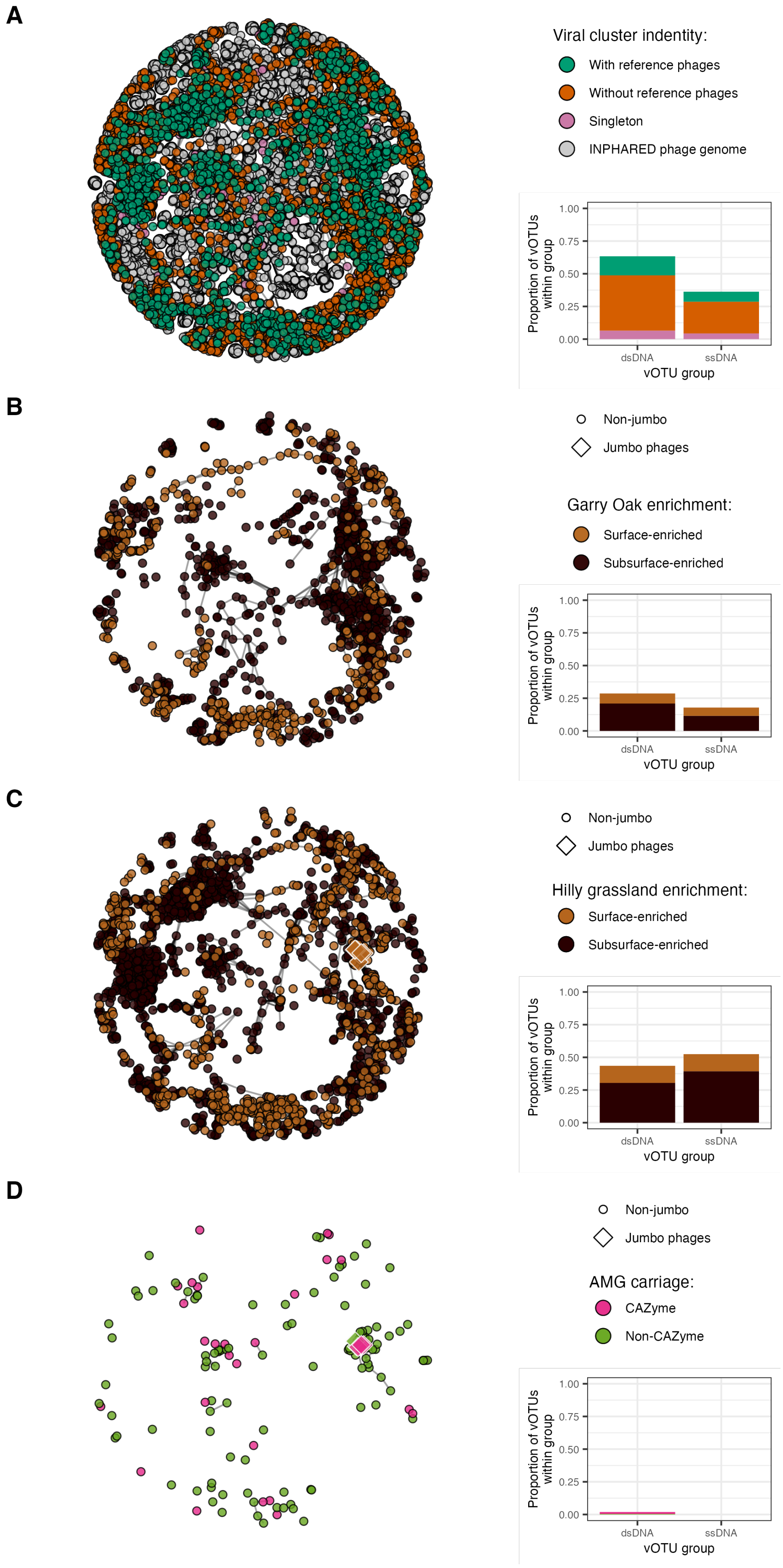

A
Viral cluster indentity:
With reference phages
Without reference phages
Singleton
INPHARED phage genome
Proportion of vOTUs within group
1.00
0.75
0.50
0.25
0.00
dsDNA
ssDNA
vOTU group
B
Non-jumbo
Jumbo phages
Garry Oak enrichment:
Surface-enriched
Subsurface-enriched
C
Hilly grassland enrichment:
D
AMG carriage:
CAZyme
Non-CAZyme

Fig. S1: Taxonomic novelty of recovered soil vOTUs. Shared protein content of recovered soil vOTUs with previously discovered phage genomes. Network graph visualisations are annotated to represent **A** viral cluster identities (6124 dsDNA vOTUs, 193 ssRNA vOTUs, and 11,600 reference genomes), **B** depth enrichment in Garry Oak (1637 dsDNA vOTUs, 19 ssDNA vOTUs), **C** depth enrichment in Hilly grassland (2820 dsDNA vOTUs, 138 ssDNA vOTUs), and **D** vOTUs carrying AMGs (152 dsDNA vOTUs, 0 ssDNA vOTUs). Bar charts (right) summarise the proportion of dsDNA vOTUs and ssDNA vOTUs included in each network visualisation. Depth enrichment represents vOTUs enriched in either surface soil (20 cm) or subsurface soil (40 cm – 115 cm).
