# Supplementary material for: Viral metagenomics reveals diverse virus-host interactions throughout the soil depth profile
Source: mBio. 2023 Nov 30;14(6):e02246-23. doi: 10.1128/mbio.02246-23 (PMC10746233; doi:10.1128/mbio.02246-23)
Supplement: Fig. S2 — Phylogenetic assessment of jumbo phage vOTUs and jumbo-related vOTUs using DNA polymerase gene. [file mbio.02246-23-s0002.pdf]

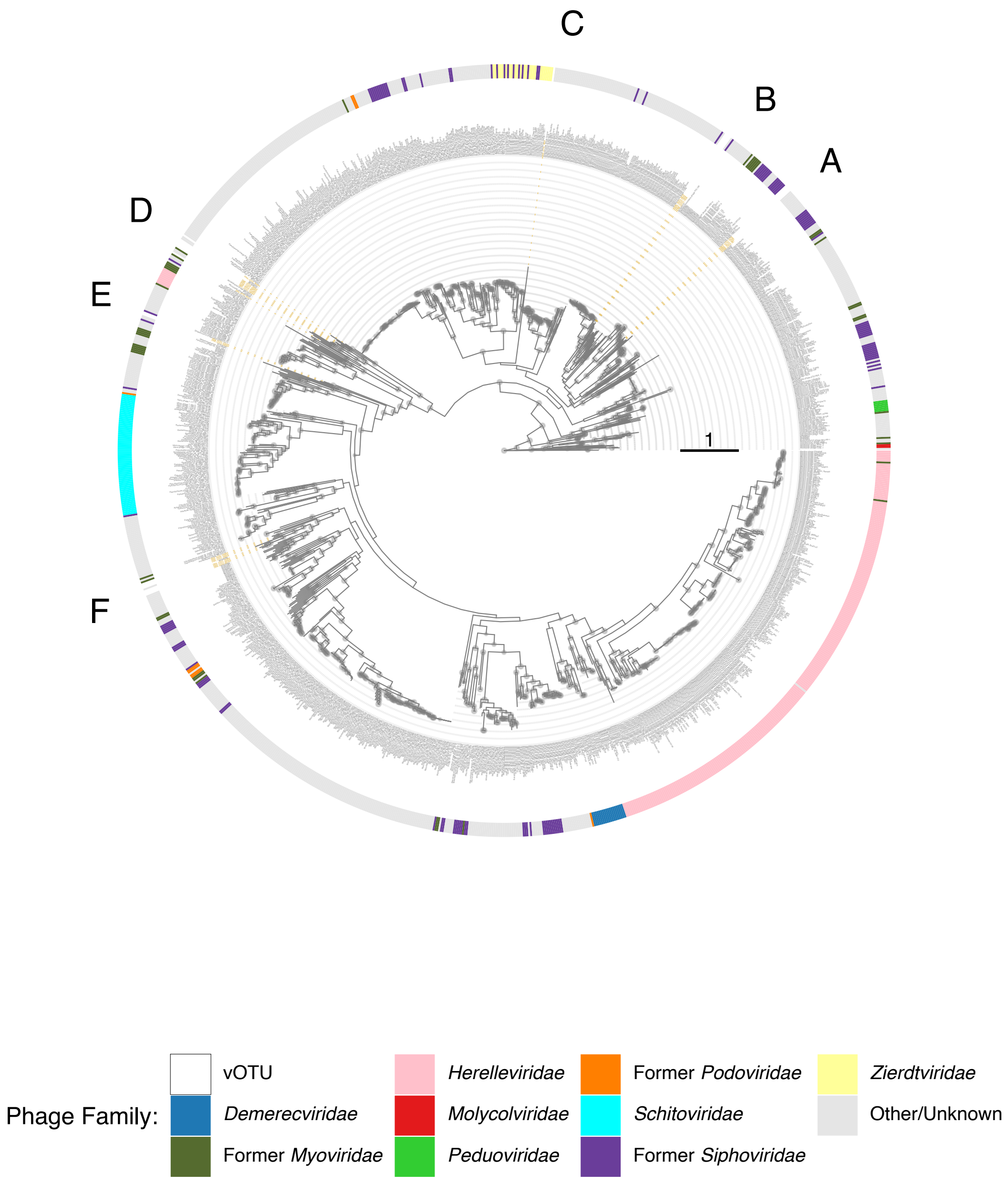
A
B
C
D
E
F
1
Phage Family:
vOTU
Demerecviridae
Former Myoviridae
Herelleviridae
Molycolviridae
Peduoviridae
Former Podoviridae
Schitoviridae
Former Siphoviridae
Zierdtviridae
Other/Unknown

Fig. S2: Phylogenetic assessment of jumbo phage vOTUs and jumbo-related vOTUs using DNA polymerase gene. Phylogeny of jumbo phage vOTUs and vOTUs sharing viral clusters with jumbo phage vOTUs (jumbo-related vOTUs) using translated DNA polymerase sequences. Phylogenetic tree contains 1284 DNA polymerase sequences from 1205 previously isolated phage sequences and 24 DNA polymerase sequences from 14 vOTUs recovered in this study (eight jumbo phage vOTUs and six jumbo-related vOTUs). Brand node labels indicate branch support: ≥ 0.9 (large circles), ≥ 0.8 (medium circles), ≥ 0.7 (small circles), < 0.7 (no circle). Tip labels indicate genome sequence name; vOTUs recovered in this study are labelled in gold. Outer ring fill colour denotes known phage families. Letters indicate the locations of 6 distinct phylogenetic groups of jumbo phage vOTUs and jumbo-related vOTUs.
