# Supplementary material for: Viral metagenomics reveals diverse virus-host interactions throughout the soil depth profile
Source: mBio. 2023 Nov 30;14(6):e02246-23. doi: 10.1128/mbio.02246-23 (PMC10746233; doi:10.1128/mbio.02246-23)
Supplement: Fig. S3 — Phylogenetic groups A, B, and F from assessment of jumbo phage vOTUs and jumbo-related vOTUs using DNA polymerase gene. [file mbio.02246-23-s0003.pdf]

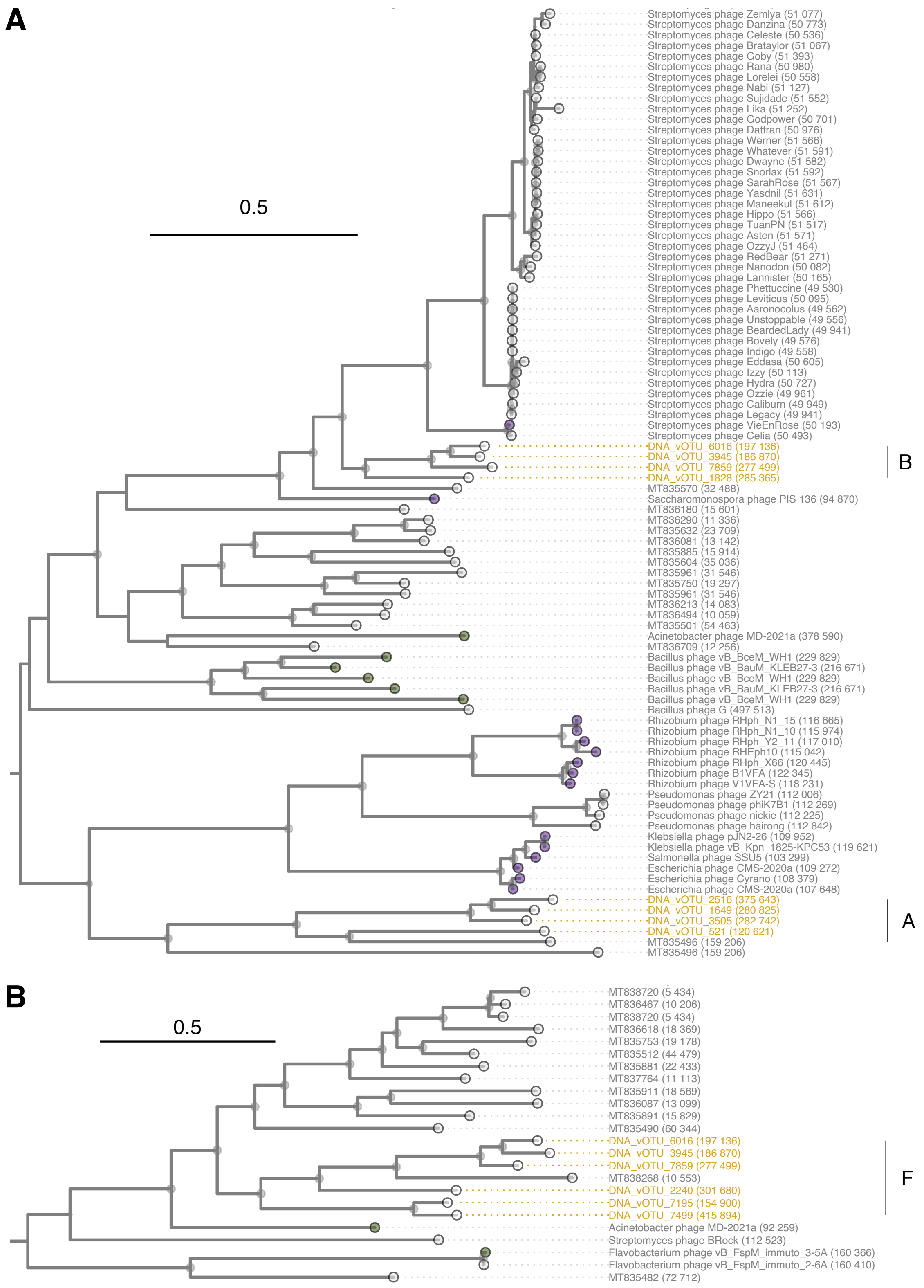
A
0.5
Streptomyces phage Zemlya (51 077)
Streptomyces phage Danzina (50 773)
Streptomyces phage Celeste (50 536)
Streptomyces phage Brataylor (51 067)
Streptomyces phage Goby (51 393)
Streptomyces phage Rana (50 980)
Streptomyces phage Lorelei (50 558)
Streptomyces phage Nabi (51 127)
Streptomyces phage Sujidade (51 552)
Streptomyces phage Lika (51 252)
Streptomyces phage Godpower (50 701)
Streptomyces phage Dattran (50 976)
Streptomyces phage Werner (51 566)
Streptomyces phage Whatever (51 591)
Streptomyces phage Dwayne (51 582)
Streptomyces phage Snorlax (51 592)
Streptomyces phage SarahRose (51 567)
Streptomyces phage Yasdnil (51 631)
Streptomyces phage Maneekul (51 612)
Streptomyces phage Hippo (51 566)
Streptomyces phage TuanPN (51 517)
Streptomyces phage Asten (51 571)
Streptomyces phage OzzyJ (51 464)
Streptomyces phage RedBear (51 271)
Streptomyces phage Nanodon (50 082)
Streptomyces phage Lannister (50 165)
Streptomyces phage Phettuccine (49 530)
Streptomyces phage Leviticus (50 095)
Streptomyces phage Aaronocolus (49 562)
Streptomyces phage Unstoppable (49 556)
Streptomyces phage BeardedLady (49 941)
Streptomyces phage Bovely (49 576)
Streptomyces phage Indigo (49 558)
Streptomyces phage Eddasa (50 605)
Streptomyces phage Izzy (50 113)
Streptomyces phage Hydra (50 727)
Streptomyces phage Ozzie (49 961)
Streptomyces phage Caliburn (49 949)
Streptomyces phage Legacy (49 941)
Streptomyces phage VieEnRose (50 193)
Streptomyces phage Celia (50 493)
DNA_vOTU_6016 (197 136)
DNA_vOTU_3945 (186 870)
DNA_vOTU_7859 (277 499)
DNA_vOTU_1828 (285 365)
B
MT835570 (32 488)
Saccharomonospora phage PIS 136 (94 870)
MT836180 (15 601)
MT836290 (11 336)
MT835632 (23 709)
MT836081 (13 142)
MT835885 (15 914)
MT835604 (35 036)
MT835961 (31 546)
MT835750 (19 297)
MT835961 (31 546)
MT836213 (14 083)
MT836494 (10 059)
MT835501 (54 463)
Acinetobacter phage MD-2021a (378 590)
MT836709 (12 256)
Bacillus phage vB_BceM_WH1 (229 829)
Bacillus phage vB_BauM_KLEB27-3 (216 671)
Bacillus phage vB_BceM_WH1 (229 829)
Bacillus phage vB_BauM_KLEB27-3 (216 671)
Bacillus phage vB_BceM_WH1 (229 829)
Bacillus phage G (497 513)
Rhizobium phage RHph_N1_15 (116 665)
Rhizobium phage RHph_N1_10 (115 974)
Rhizobium phage RHph_Y2_11 (117 010)
Rhizobium phage RHEph10 (115 042)
Rhizobium phage RHph_X66 (120 445)
Rhizobium phage B1VFA (122 345)
Rhizobium phage V1VFA-S (118 231)
Pseudomonas phage ZY21 (112 006)
Pseudomonas phage phiK7B1 (112 269)
Pseudomonas phage nickie (112 225)
Pseudomonas phage hairong (112 842)
Klebsiella phage pJN2-26 (109 952)
Klebsiella phage vB_Kpn_1825-KPC53 (119 621)
Salmonella phage SSU5 (103 299)
Escherichia phage CMS-2020a (109 272)
Escherichia phage Cyrano (108 379)
Escherichia phage CMS-2020a (107 648)
DNA_vOTU_2516 (375 643)
DNA_vOTU_1649 (280 825)
DNA_vOTU_3505 (282 742)
DNA_vOTU_521 (120 621)
A
MT835496 (159 206)
MT835496 (159 206)
B
0.5
MT838720 (5 434)
MT836467 (10 206)
MT838720 (5 434)
MT836618 (18 369)
MT835753 (19 178)
MT835512 (44 479)
MT835881 (22 433)
MT837764 (11 113)
MT835911 (18 569)
MT836087 (13 099)
MT835891 (15 829)
MT835490 (60 344)
DNA_vOTU_6016 (197 136)
DNA_vOTU_3945 (186 870)
DNA_vOTU_7859 (277 499)
MT838268 (10 553)
DNA_vOTU_2240 (301 680)
DNA_vOTU_7195 (154 900)
DNA_vOTU_7499 (415 894)
F
Acinetobacter phage MD-2021a (92 259)
Streptomyces phage BRock (112 523)
Flavobacterium phage vB_FspM_immuto_3-5A (160 366)
Flavobacterium phage vB_FspM_immuto_2-6A (160 410)
MT835482 (72 712)

Fig. S3: Phylogenetic groups A, B, and F from assessment of jumbo phage vOTUs and jumbo-related vOTUs using DNA polymerase gene. Further investigation of distinct phylogenetic groups identified from Fig. S2: **A** Groups A and B, **B** Group F. Brand node labels indicate branch support: ≥ 0.9 (large circles), ≥ 0.8 (medium circles), ≥ 0.7 (small circles), < 0.7 (no circle). Tip node fill colour denotes known phage families. Tip labels indicate genome sequence name and genome length in bp; vOTUs recovered in this study are labelled in gold. Letters indicate the locations of distinct phylogenetic groups of jumbo phage vOTUs and jumbo-related vOTUs.
