# Supplementary material for: Viral metagenomics reveals diverse virus-host interactions throughout the soil depth profile
Source: mBio. 2023 Nov 30;14(6):e02246-23. doi: 10.1128/mbio.02246-23 (PMC10746233; doi:10.1128/mbio.02246-23)
Supplement: Fig. S4 — Distribution of microbial populations throughout soil depth profiles. [file mbio.02246-23-s0004.pdf]

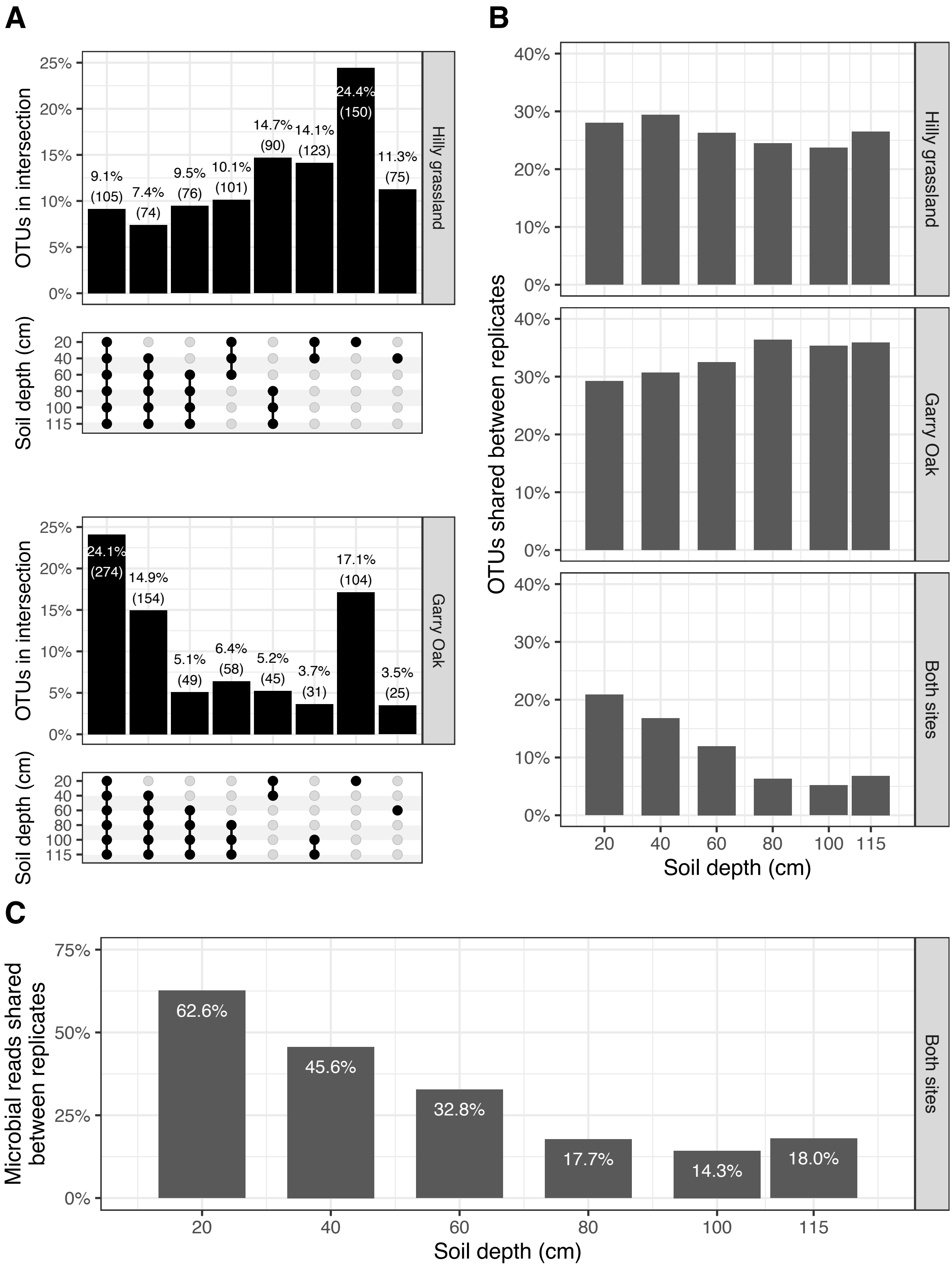

A
B
C
OTUs in intersection
Hilly grassland
Garry Oak
Both sites
Soil depth (cm)
OTUs shared between replicates
Microbial reads shared between replicates
9.1% (105)
7.4% (74)
9.5% (76)
10.1% (101)
14.7% (90)
14.1% (123)
24.4% (150)
11.3% (75)
24.1% (274)
14.9% (154)
5.1% (49)
6.4% (58)
5.2% (45)
3.7% (31)
17.1% (104)
3.5% (25)
62.6%
45.6%
32.8%
17.7%
14.3%
18.0%

Fig. S4: Distribution of microbial populations throughout soil depth profiles. A Overlap in the detection of microbial populations between different depths of each site. Intersection matrix denotes depths at which OTUs were detected. Bar plot displays the percentage of total OTUs detected in depth intersection. Bars shown for depth intersections with the eight most OTUs detected. **B** Overlap in the detection of microbial populations between the same depths (i.e., between replicates) of each site and both sites combined. **C** Percentage of microbial abundance shared between the same depths (i.e., between replicates) of both sites combined.
