# Supplementary material for: Viral metagenomics reveals diverse virus-host interactions throughout the soil depth profile
Source: mBio. 2023 Nov 30;14(6):e02246-23. doi: 10.1128/mbio.02246-23 (PMC10746233; doi:10.1128/mbio.02246-23)
Supplement: Fig. S5 — Correlation of viral community and microbial community. [file mbio.02246-23-s0005.pdf]

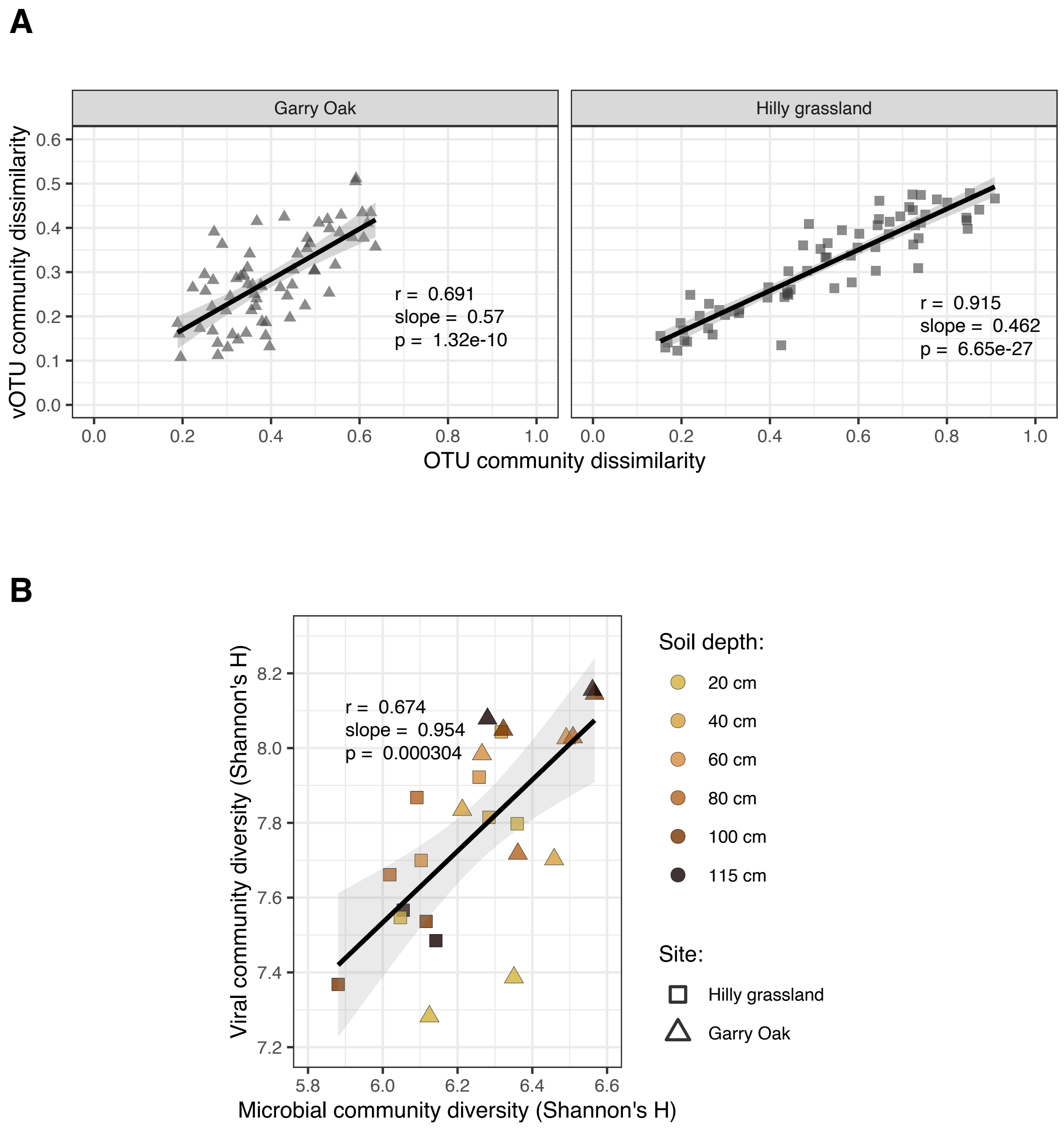
A
Garry Oak
Hilly grassland
vOTU community dissimilarity
0.0
0.1
0.2
0.3
0.4
0.5
0.6
r = 0.691
slope = 0.57
p = 1.32e-10
r = 0.915
slope = 0.462
p = 6.65e-27
0.0
0.2
0.4
0.6
0.8
1.0
OTU community dissimilarity
B
Viral community diversity (Shannon's H)
7.2
7.4
7.6
7.8
8.0
8.2
r = 0.674
slope = 0.954
p = 0.000304
5.8
6.0
6.2
6.4
6.6
Microbial community diversity (Shannon's H)
Soil depth:
20 cm
40 cm
60 cm
80 cm
100 cm
115 cm
Site:
Hilly grassland
Garry Oak

Fig. S5: Correlation of viral community and microbial community A structure and **B** diversity. Microbial community described using OTU abundances. Trend lines represent linear regression estimates, with shaded cloud representing 95% confidence interval. r corresponds to Pearson's correlation coefficient and p corresponds to the associated p-value. Shapes indicate site: Hilly grassland (squares) and Garry Oak (triangles). Shapes are coloured based on soil depth.
