# Supplementary material for: Viral metagenomics reveals diverse virus-host interactions throughout the soil depth profile
Source: mBio. 2023 Nov 30;14(6):e02246-23. doi: 10.1128/mbio.02246-23 (PMC10746233; doi:10.1128/mbio.02246-23)
Supplement: Fig. S6 — Correlation of viral abundances and host abundances. [file mbio.02246-23-s0006.pdf]

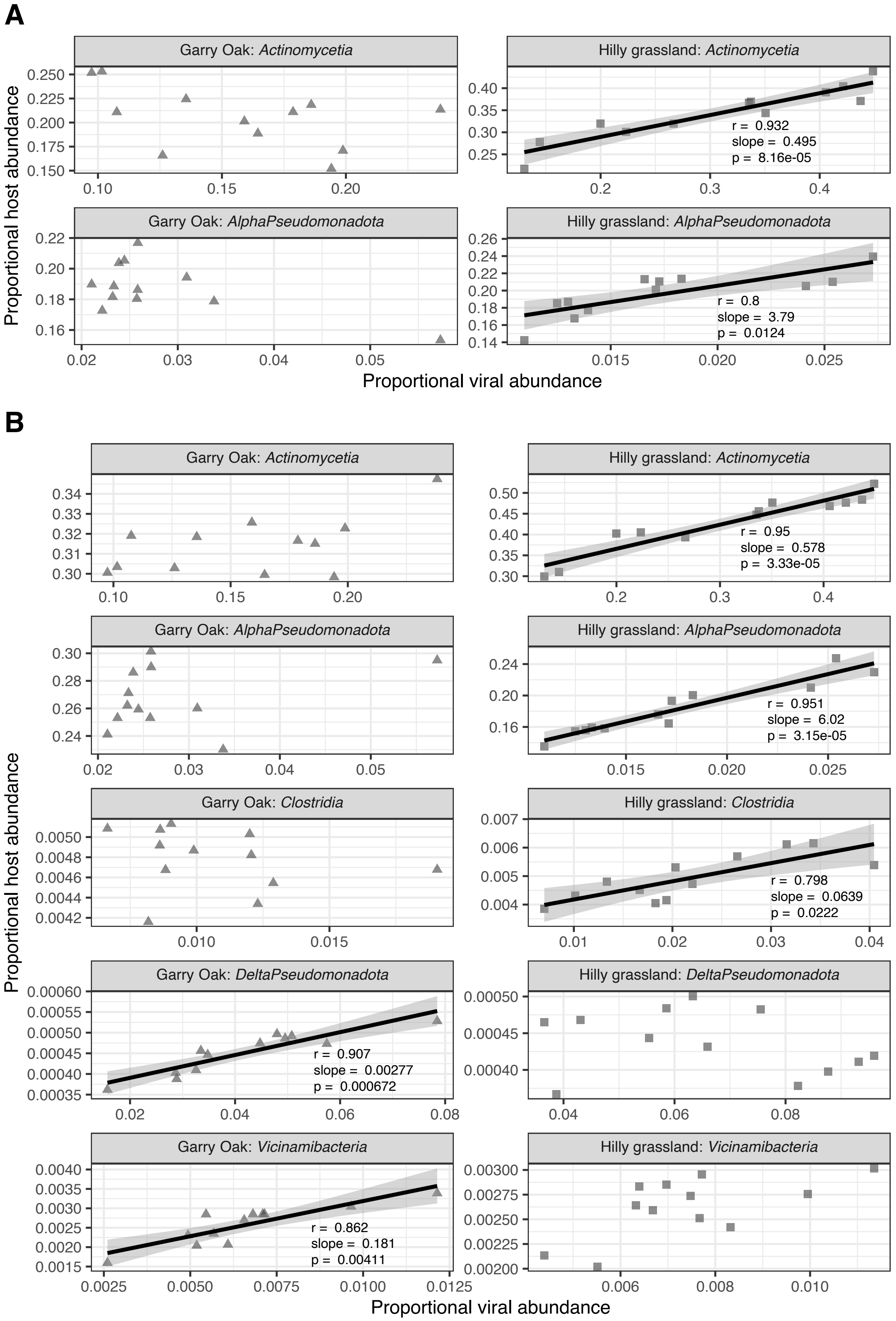
A
Garry Oak: *Actinomycetia*
Hilly grassland: *Actinomycetia*
r = 0.932
slope = 0.495
p = 8.16e-05
Garry Oak: *AlphaPseudomonadota*
Hilly grassland: *AlphaPseudomonadota*
r = 0.8
slope = 3.79
p = 0.0124
Proportional host abundance
Proportional viral abundance
B
Garry Oak: *Actinomycetia*
Hilly grassland: *Actinomycetia*
r = 0.95
slope = 0.578
p = 3.33e-05
Garry Oak: *AlphaPseudomonadota*
Hilly grassland: *AlphaPseudomonadota*
r = 0.951
slope = 6.02
p = 3.15e-05
Garry Oak: *Clostridia*
Hilly grassland: *Clostridia*
r = 0.798
slope = 0.0639
p = 0.0222
Garry Oak: *DeltaPseudomonadota*
r = 0.907
slope = 0.00277
p = 0.000672
Hilly grassland: *DeltaPseudomonadota*
Garry Oak: *Vicinamibacteria*
r = 0.862
slope = 0.181
p = 0.00411
Hilly grassland: *Vicinamibacteria*
Proportional host abundance
Proportional viral abundance

**Fig. S6: Correlation of viral abundances and host abundances using A** OTU abundance and **B** raw read taxonomy**.** Bacterial classes are only shown if a significant relationship with depth was observed in at least one of the sites. Trend line represents linear regression estimates, with shaded cloud representing 95% confidence interval. $r$ corresponds to Pearson's correlation coefficient and $p$ corresponds to the associated p-value, corrected for multiple comparisons with the Holm algorithm.
