# Supplementary figures and images for: Viral metagenomics reveals diverse virus-host interactions throughout the soil depth profile
Source: mBio. 2023 Nov 30;14(6):e02246-23. doi: 10.1128/mbio.02246-23 (PMC10746233; doi:10.1128/mbio.02246-23)

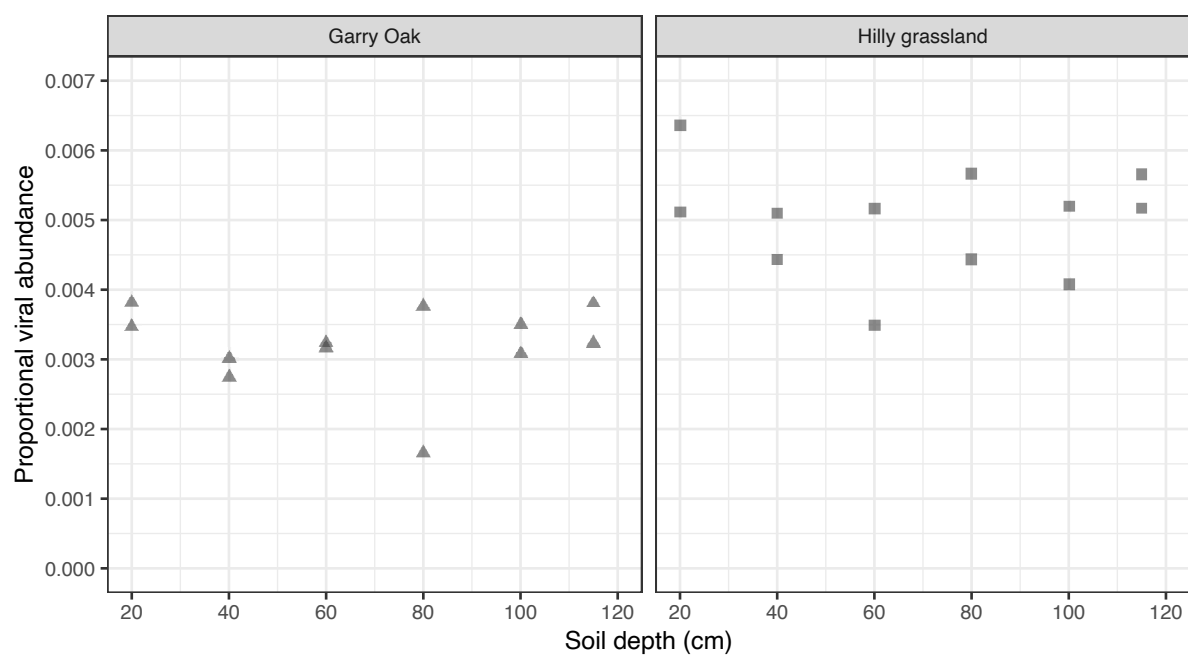

**Fig. S7: Relative abundance of viruses carrying carbohydrate-active enzymes.**

Supplement: Fig. S7 — Relative abundance of viruses carrying carbohydrate-active enzymes. [file mbio.02246-23-s0007.pdf]
