# Supplementary material for: Viral metagenomics reveals diverse virus-host interactions throughout the soil depth profile
Source: mBio. 2023 Nov 30;14(6):e02246-23. doi: 10.1128/mbio.02246-23 (PMC10746233; doi:10.1128/mbio.02246-23)
Supplement: Fig. S8 — Genome maps of high-quality viral genomes carrying ABC transporters under positive selection. [file mbio.02246-23-s0008.pdf]

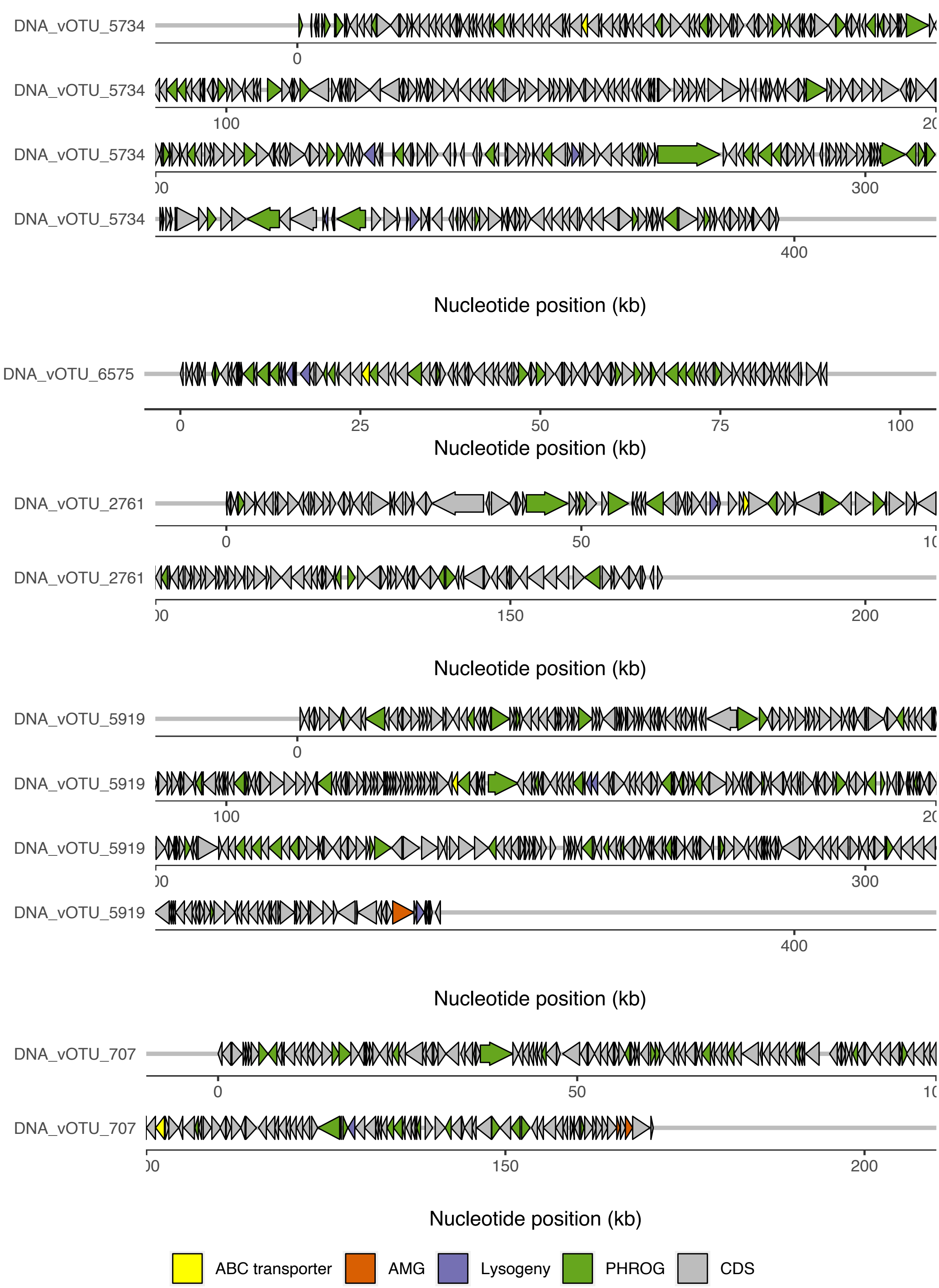

DNA_vOTU_5734
0
DNA_vOTU_5734
100
DNA_vOTU_5734
300
DNA_vOTU_5734
400
Nucleotide position (kb)
DNA_vOTU_6575
0
25
50
75
100
Nucleotide position (kb)
DNA_vOTU_2761
0
50
DNA_vOTU_2761
150
200
Nucleotide position (kb)
DNA_vOTU_5919
0
DNA_vOTU_5919
100
DNA_vOTU_5919
300
DNA_vOTU_5919
400
Nucleotide position (kb)
DNA_vOTU_707
0
50
DNA_vOTU_707
150
200
Nucleotide position (kb)
ABC transporter
AMG
Lysogeny
PHROG
CDS

Fig. S8: Genome maps of high-quality viral genomes carrying ABC transporters under positive selection. Arrow fill colour indicates gene function.
