# Supplementary material for: Viral metagenomics reveals diverse virus-host interactions throughout the soil depth profile
Source: mBio. 2023 Nov 30;14(6):e02246-23. doi: 10.1128/mbio.02246-23 (PMC10746233; doi:10.1128/mbio.02246-23)
Supplement: Fig. S9 — Rank abudance of jumbo phages and viruses carrying CAZymes. [file mbio.02246-23-s0009.pdf]

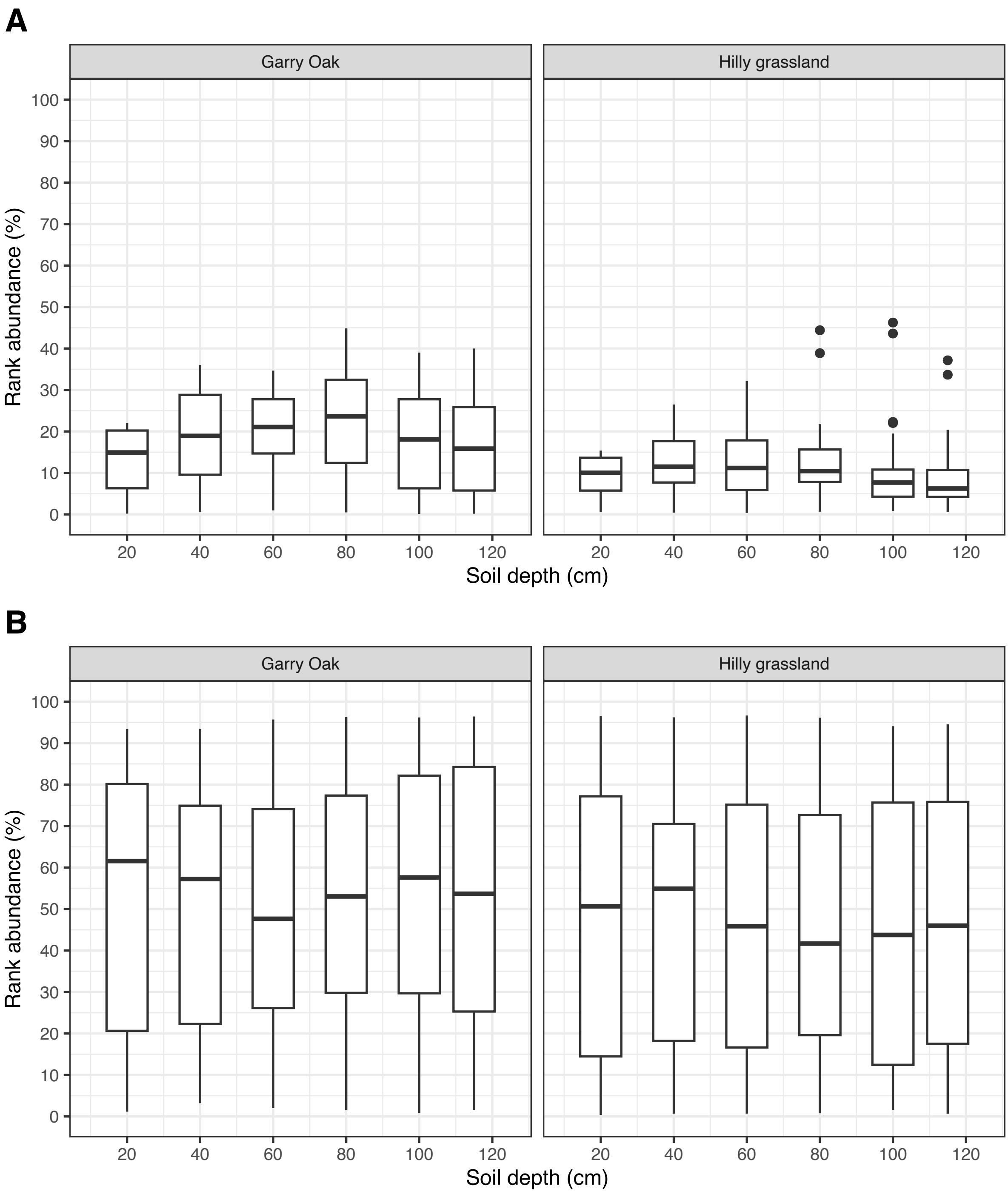
A
Garry Oak
Hilly grassland
Rank abundance (%)
0
10
20
30
40
50
60
70
80
90
100
20
40
60
80
100
120
Soil depth (cm)
B
Garry Oak
Hilly grassland
Rank abundance (%)
Soil depth (cm)

Fig. S9: Rank abundance of A jumbo phages and **B** viruses carrying CAZymes. Rank abundance represented as a percentage of 10,196 vOTUs. 0% indicates the lowest rank and the most abundant vOTU, while 100% indicates the highest rank and the least abundant vOTU. Boxes denote median, upper, and lower quartiles. Whiskers indicate minimal and maximal values, with outliers in filled circles.
