# Supplementary material for: Viral metagenomics reveals diverse virus-host interactions throughout the soil depth profile
Source: mBio. 2023 Nov 30;14(6):e02246-23. doi: 10.1128/mbio.02246-23 (PMC10746233; doi:10.1128/mbio.02246-23)
Supplement: Fig. S10 — Relative abundance of viruses carrying auxiliary metabolic genes by host class. [file mbio.02246-23-s0010.pdf]

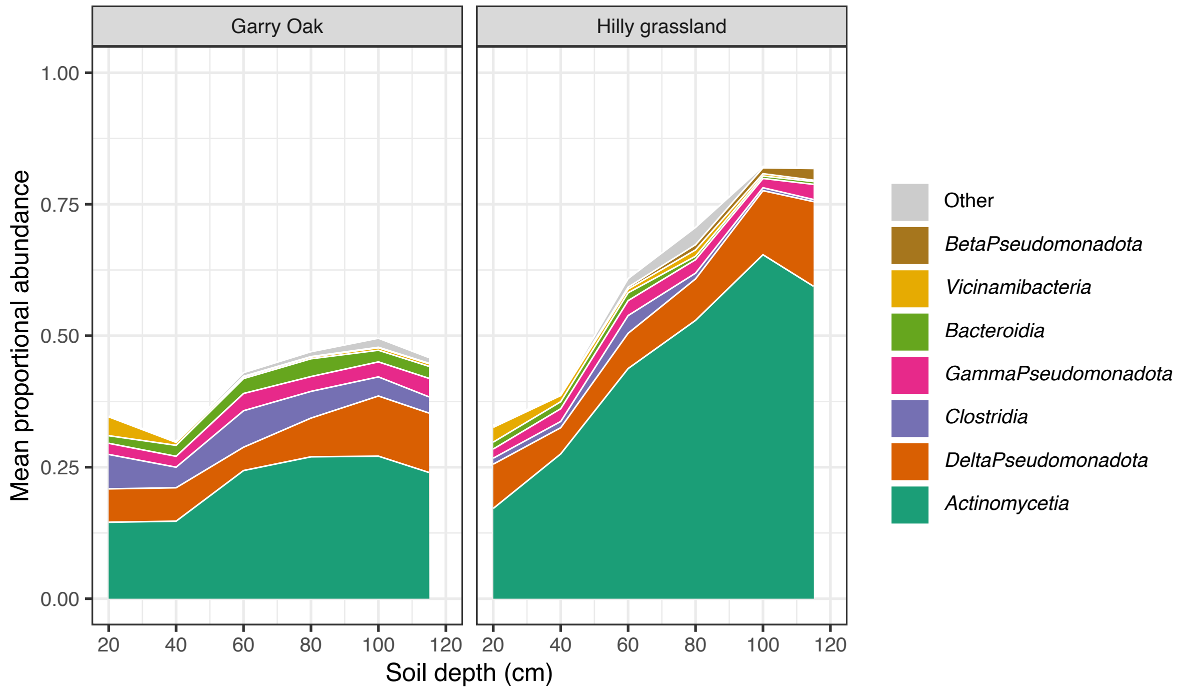
Garry Oak
Hilly grassland
Mean proportional abundance
1.00
0.75
0.50
0.25
0.00
20
40
60
80
100
120
Soil depth (cm)
Other
BetaPseudomonadota
Vicinamibacteria
Bacteroidia
GammaPseudomonadota
Clostridia
DeltaPseudomonadota
Actinomycetia

Fig. S10: Relative abundance of viruses carrying auxiliary metabolic genes by host class. Proportional abundance of vOTUs carrying AMGs plotted across soil depth. Fill colour indicates host class.
